# A targetable ‘rogue’ neutrophil-subset, [CD11b+DEspR+] immunotype, is associated with severity and mortality in acute respiratory distress syndrome (ARDS) and COVID-19-ARDS

**DOI:** 10.1038/s41598-022-09343-1

**Published:** 2022-04-04

**Authors:** Victoria L. M. Herrera, Allan J. Walkey, Mai Q. Nguyen, Christopher M. Gromisch, Julie Z. Mosaddhegi, Matthew S. Gromisch, Bakr Jundi, Soeren Lukassen, Saskia Carstensen, Ridiane Denis, Anna C. Belkina, Rebecca M. Baron, Mayra Pinilla-Vera, Meike Mueller, W. Taylor Kimberly, Joshua N. Goldstein, Irina Lehmann, Angela R. Shih, Roland Eils, Bruce D. Levy, Nelson Ruiz-Opazo

**Affiliations:** 1 Whitaker Cardiovascular Institute and Department of Medicine, Boston University School of Medicine, Boston, MA USA; 2 Section of Pulmonary and Critical Care, Department of Medicine, Boston University School of Medicine, and Boston Medical Center, Boston, MA USA; 3 Pulmonary and Critical Care Medicine, Department of Medicine, Brigham and Women’s Hospital, Harvard Medical School, Boston, MA USA; 4 Center for Digital Health, Berlin Institute of Health and Charité - Universitätsmedizin Berlin, Corporate Member of Freie Universität Berlin, Humboldt-Universität Zu Berlin, Berlin, Germany; 5 Fraunhofer Institute for Toxicology and Experimental Medicine, Hannover, Germany; 6 General Clinical Research Center, Boston University School of Medicine, Boston, MA USA; 7 Department of Pathology and Laboratory Medicine, Flow Cytometry Core Facility, Boston University School of Medicine, Boston, MA USA; 8 Division of Neurocritical Care, Massachusetts General Hospital, Harvard Medical School, Boston, MA USA; 9 Department of Emergency Medicine, Massachusetts General Hospital, Harvard Medical School, Boston, MA USA; 10 Molecular Epidemiology Unit, Charité - Universitätsmedizin Berlin, Corporate Member of Freie Universität Berlin, Humboldt-Universität Zu Berlin and Berlin Institute of Health (BIH), Berlin, Germany; 11 Department of Pathology, Massachusetts General Hospital, Harvard Medical School, Boston, MA USA

**Keywords:** Respiratory tract diseases, Translational research

## Abstract

Neutrophil-mediated secondary tissue injury underlies acute respiratory distress syndrome (ARDS) and progression to multi-organ-failure (MOF) and death, processes linked to COVID-19-ARDS. This secondary tissue injury arises from dysregulated neutrophils and neutrophil extracellular traps (NETs) intended to kill pathogens, but instead cause cell-injury. Insufficiency of pleiotropic therapeutic approaches delineate the need for inhibitors of dysregulated neutrophil-subset(s) that induce subset-specific apoptosis critical for neutrophil function-shutdown. We hypothesized that neutrophils expressing the pro-survival dual endothelin-1/VEGF-signal peptide receptor, DEspR, are apoptosis-resistant like DEspR+ cancer-cells, hence comprise a consequential pathogenic neutrophil-subset in ARDS and COVID-19-ARDS. Here, we report the significant association of increased peripheral DEspR+CD11b+ neutrophil-counts with severity and mortality in ARDS and COVID-19-ARDS, and intravascular NET-formation, in contrast to DEspR[-] neutrophils. We detect DEspR+ neutrophils and monocytes in lung tissue patients in ARDS and COVID-19-ARDS, and increased neutrophil RNA-levels of DEspR ligands and modulators in COVID-19-ARDS scRNA-seq data-files. Unlike DEspR[-] neutrophils, DEspR+CD11b+ neutrophils exhibit delayed apoptosis, which is blocked by humanized anti-DEspR-IgG4^S228P^ antibody, hu6g8, in ex vivo assays. Ex vivo live-cell imaging of *Rhesus-derived* DEspR+CD11b+ neutrophils showed hu6g8 target-engagement, internalization, and induction of apoptosis. Altogether, data identify DEspR+CD11b+ neutrophils as a targetable ‘rogue’ neutrophil-subset associated with severity and mortality in ARDS and COVID-19-ARDS.

## Introduction

Acute respiratory distress syndrome (ARDS) and progression to multi-organ failure (MOF) comprise a pathological spectrum of secondary ‘bystander’ tissue injury arising when one’s inflammatory response to an inciting ‘primary injury’ – be it infectious or non-infectious—becomes dysregulated and excessive^1^. Stopping this feed-forward secondary tissue injury in ARDS and MOF remains an important unmet need, as there is no FDA-approved pharmacotherapy able to reduce the high mortality in ARDS from MOF^2^. The lethality of secondary tissue injury is highlighted by the COVID-19 pandemic as progression to ARDS and multi-organ failure are accelerated, and comprise the major cause of death in severe COVID-19^3^.

To address the unmet need for therapies for ARDS and COVID-19-ARDS, we reasoned that identification of pathogenic commonalities in ARDS and COVID-19-ARDS could help identify novel therapeutic targets that attenuate ARDS-MOF. Neutrophils have long been implicated in ARDS-MOF^1,4,5^, through neutrophil-mediated microvascular endothelial injury, capillary permeability^6^, and neutrophil-extracellular trap (NET)-associated multi-organ endothelial and lung epithelial injury^7^. More recently, the association of increased neutrophil–lymphocyte ratios (NLR) with ARDS^8^ and COVID-19-ARDS^9^ severity and poor prognosis, and increased bloodstream levels of NETs-associated myeloperoxidase-DNA complexes in COVID-19-ARDS^10,11^, further support the central role of neutrophils in ARDs-MOF. Additionally, cumulative comparative single cell RNA-sequencing (scRNA-seq) analysis of mild and critically ill COVID-19-patients, and non-infected healthy controls, provide molecular evidence supporting the central role of neutrophils in severe COVID-19^12–15^.

However, inhibiting neutrophils to mitigate ARDS-MOF severity and mortality has been elusive despite preclinical efficacy in animal models of acute lung injury^4^. The cumulative low translatability, due most likely to species differences in neutrophil biology and to multifactorial complexities in ARDS and COVID-19-ARDS pathogeneses not recapitulated in corresponding preclinical models that spontaneously recover, provides scientific basis for ex vivo analysis of ARDS and severe COVID-19-ARDS patient whole blood samples. Moreover, given putative heterogeneity in ARDS (endotypes)^1,16^ and neutrophils (subsets)^1,17^, prospective ex vivo studies of patient neutrophils and NET-forming neutrophils can provide key insight(s) into putative neutrophil-subset(s) in progression of MOF in ARDS and COVID-19-ARDS.

Based on insights from the study of DEspR+ cancer stem-like cells (CSCs) exhibiting aberrant apoptosis-resistance associated with myeloid cell leukemia (Mcl1) levels, a key apoptosis-evasion protein in cancer^18^, we reasoned that DEspR+ neutrophils would also have survival advantages as Mcl1 levels correlate with neutrophil survival^19^. Since neutrophil apoptosis is required for efferocytosis, function shutdown and active resolution of inflammation^20^, longer neutrophil survival or delayed ‘constitutive apoptosis’ increases risk for dysregulation and subsequent neutrophil-mediated tissue injury. Additionally, given that endothelin-1 (ET1) levels, one of two DEspR ligands^21^, are elevated in ARDS^22^, and since ET1 is known to enhance neutrophil activation and functionality^23^, ET1-mediated DEspR activation could play key pathogenic role(s) in neutrophil-mediated secondary tissue injury in ARDS.

We therefore tested the unifying hypothesis that DEspR+ neutrophils comprise an activated neutrophil-subset with pathogenic survival advantage over DESpR[-] activated neutrophils, and whose cumulative increase would contribute to MOF, hence correlate with severity and/or mortality in ARDS and COVID-19-ARDS. Here, we studied (1) whether DEspR+ neutrophils comprise a neutrophil-subset associated with ARDS and COVID-19-ARDS severity or mortality, (2) whether identification of the DEspR+ neutrophil-subset is reproducible in different research labs and concordant with scRNA-seq findings in severe COVID-19, and (3) whether DEspR+ neutrophils can be safely inhibited as a potential therapeutic target on dysregulated neutrophils.

## Results

### Identification of DEspR+ human neutrophils in 24-h old NHV whole blood

To determine DEspR-expression on human neutrophils as a potential subset-marker, we analyzed DEspR protein levels by immunocytology and western blot analysis. First, we performed immunocytology of 24-h (hours) old normal human volunteer (NHV) neutrophils. We used a recombinant, humanized anti-DEspR antibody, cross reactive to human, monkey and rodent DEspR, with a hinge-stabilized [S228P]IgG4 backbone, hu6g8, developed by us and validated for detection of DEspR-specific cell expression, and in vitro and in vivo DEspR-inhibition resulting in apoptosis in DEspR+ tumor cells and cancer stem-like cells^18^.

**Figure 1:**
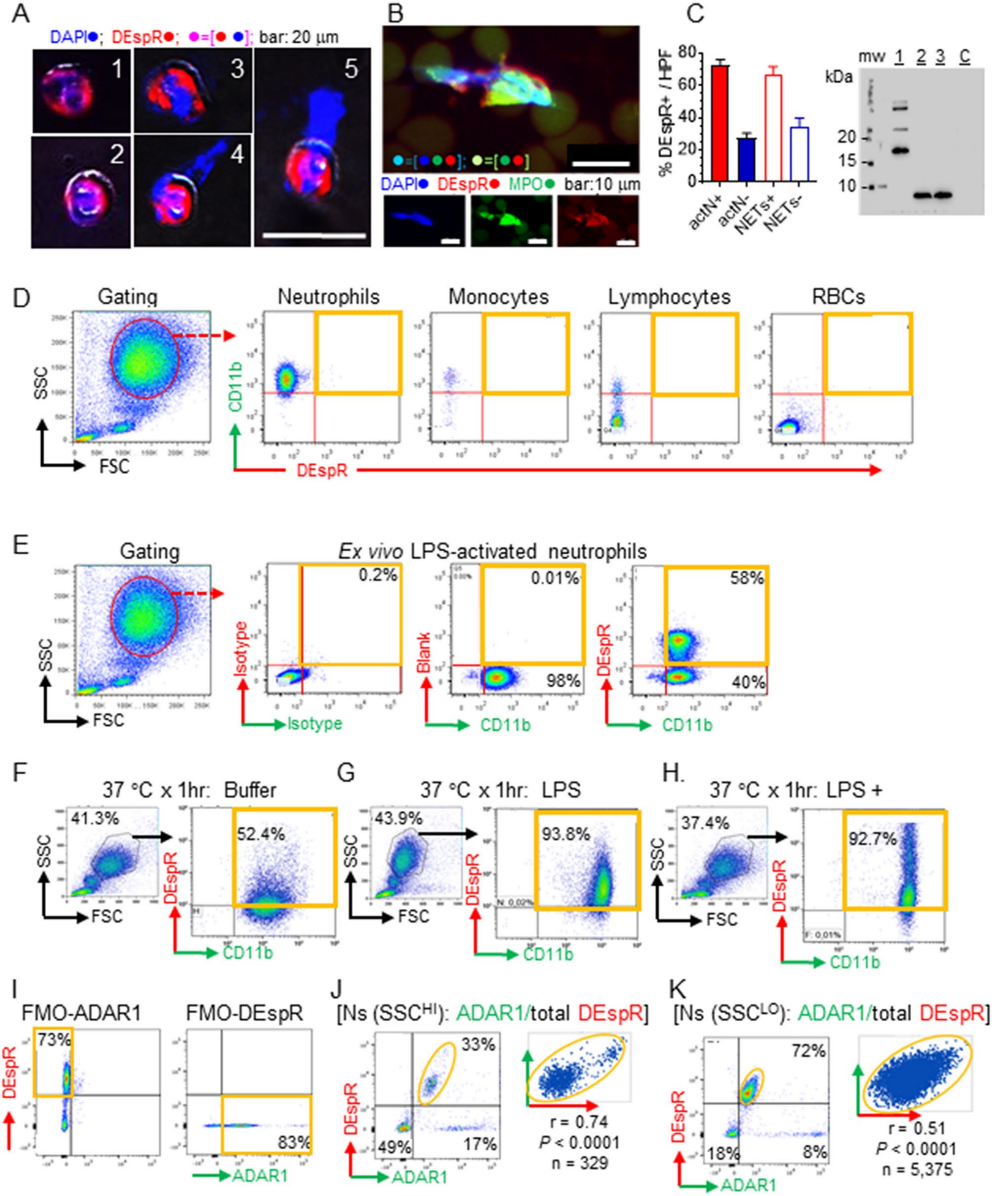
DEspR expression on normal human volunteer (NHV) neutrophils. (**A**) Representative immunofluorescence-staining of DEspR+ expression in > 24-h old surviving NHV neutrophils with: #1: polylobulated nucleus; #2: altered nucleus with DNA-rimming towards cell membrane; #3: decondensing nucleus and some extrusion; #4 and #5: NET-forming neutrophils with extruded DNA, residual nuclei and intact cell membrane. Panel: hu6g8-AF568 DEspR+ red circle, DAPI blue circle, merged pink circle; Bar = 20 μm. (See Supplementary Fig. S1A). (**B**) Immunofluorescence of a representative extruded neutrophil extracellular trap (NET) exhibiting DAPI+ DNA with MPO+ and DEspR+ components. Bar = 10 μm. (**C**) Left: bar graph showing semi-quantitation of % DEspR+ neutrophils and NET-forming neutrophils in 6 high power fields per slide in 3 slides prepared from NHV polled neutrophils. Right: Western blot analysis showing DEspR expressed in normal human kidney (HK), neutrophils (HN1) and LPS-stimulated neutrophils (HN2), MW, molecular weight markers. LB, Laemli buffer as negative control. (**D**) Flow cytometry (FCM) analysis of EDTA-anticoagulated NHV whole blood, non-stimulated. Gating of white blood cells for neutrophils, monocytes, lymphocytes and red blood cells (RBC) by their respective clouds determined by differential forward scatter (FSC, x-axis), side scatter (SSC, Y-axis).features, and fluorescence markers: anti-CD11b-FITC, anti-DEspR (hu6g8)-AF568. (**E**) FCM analysis of NHV (EDTA-anticoagulated) whole blood stimulated with LPS (75 μg/ml, 1-h at 37 °C), Panels left-to-right: FSC/SSC gating for neutrophils; isotype controls hIgG4-AF568, mIgG2-AF488; FMO-DEspR); CD11b (X-axis) vs DEspR (Y = axis). (**F–H**) FCM analysis of NHV (Li-heparin anticoagulated) showing increased DEspR+ CD11b+ neutrophils in 3 different conditions: (**F**) after 1-h incubation at 37 °C, (**G**) after 1 h LPS-stimulation (75 ng/ml) at 37 °C; and (**H**) after paraformaldehyde permeabilization done after 1 h LPS-incubation at 37 °C. PFA-permeabilization detects increased DEspR with high (intracellular and cell-surface DEspR) and low (cell-surface) DEspR+ expression. (**I-K**) Correlation analysis of intracellular ADAR1 and total DEspR (surface and intracellular) in fixed, permeabilized neutrophils. (**I**) Fluorescence minus one (FMO)-ADAR1 and FMO-DEspR controls to set fluorescence gates after two neutrophil are gated by SSC and FSC; (**J**) Fixed permeabilized neutrophil subset with low FSC, high SSC; Q1: subtracting isotype background, 0% DEspR+ ADAR1[−]; Q2: 100% of DEspR+ neutrophils are ADAR1+, with 0.74 Pearson correlation coefficient, P < 0.0001, n = 329. Q3: ADAR1+/DEspR[−] neutrophils; Q4: DEspR[−]ADAR1[−]. (**K**) Fixed permeabilized neutrophil subset with low FSC, low SSC; subtracting isotype background 100% of DEspR+ neutrophils in quadrant Q2 are ADAR1+, with 0.51 Pearson correlation coefficient, P < 0.0001, n = 5,375.

Direct immunofluorescence (IF)-staining of 24-h old NHV-neutrophils detected predominantly DEspR-positive (DEspR +) neutrophil nuclei, cell membrane and cytoplasm (Fig. 1A, Supplementary Fig. S1A). IF-staining also detected DEspR+ neutrophils with different degrees of extruded DNA and intact cell membranes (Fig. 1A), concordant with early cytolytic NET-formation or non-cytolytic neutrophil-NET formation with mitochondrial (mt)DNA^24,25^. We also detected classical cytolytic NET-formation with extruded myeloperoxidase-positive (MPO +) DNA-cloud and disrupted cell membranes^25^, and unexpectedly, also DEspR+ (Fig. 1B). Majority of 24-h old neutrophils and NET-forming neutrophils were DEspR+ in these ex vivo experimental conditions (Fig. 1C).

Western blot analysis of whole cell protein isolates from the same 24-h old NHV neutrophils detected the expected size DEspR protein, thus confirming DEspR+ expression. Western blot analysis also detected a larger DEspR protein in human kidney (Fig. 1C, Supplementary Fig. S1A) due to N-glycosylation as shown previously in cancer cells^26^.

### Identification of DEspR+ CD11b+ neutrophil-subset in LPS-TLR4 activated NHV neutrophils

To further assess DEspR+ neutrophil subtype, while also validating flow cytometry analysis protocols for ex vivo studies of ARDS patient whole blood samples, we performed flow cytometry (FCM) double-immuno-phenotyping for cell-surface co-expression of DEspR and CD11b. We selected CD11b as a marker of activated neutrophils as CD11b mediates neutrophil-complement system crosstalk, and since CD11b+ neutrophils are increased in ARDS patient peripheral blood and broncho-alveolar fluid^17^. Flow cytometry analysis of NHV whole blood (EDTA-anticoagulated) samples detected minimal, if any, DEspR+ expression on the cell surface of intact CD11b+ neutrophils, monocytes and lymphocytes in baseline conditions, and no expression on red blood cells (RBCs) (Fig. 1D). In keeping with known neutrophil functions, DEspR expression was increased by standard RBC-lysis step when done before antibody binding (Supplementary Fig. S1B), likely in response to damage associated molecular patterns (DAMPs) released during RBC-lysis^27^. DEspR expression also increased with time from blood sampling greater than 1-h, whether at 4 °C or at 37 °C (Supplementary Fig. S1C).

As toll-like receptor 4 (TLR4) is a cell-surface receptor on neutrophils activated by DAMPs released by cell injury, as in RBC-lysis, or pathogen-associated molecular patterns (PAMPS), we tested whether TLR4-activation by bacterial lipopolysaccharide (LPS), a PAMP, would increase DEspR+ neutrophil levels in NHV-whole blood. After exposure to LPS, flow cytometry detected increased DEspR+ expression in majority, but not all, CD11b+ neutrophils (Fig. 1E). Upregulation of DEspR+CD11b+ neutrophils by LPS in vitro is concordant with TLR4-activation by DAMPs^28^, as well as PAMPS, in both ARDS^29^ and in COVID-19-ARDS^14^, and characterized by increased CD11b+ expression on neutrophils^30^.

### Multi-center verification of DEspR+ CD11b+ neutrophil-subset and protocol optimization

To independently test detection of DEspR+CD11b+ neutrophils in NHV blood samples, collaborators performed flow cytometry analysis on Li-heparin anticoagulated NHV whole blood that would allow more robust neutrophil-activation without EDTA-chelation of Ca^2+^and Mg^+2^ ions. As expected, testing of Li-heparin anticoagulated NHV whole blood detected higher DEspR+CD11b+ activated neutrophils: ~ 52.4% in 37 °C 1-h incubation in buffer (Fig. 1F), and ~ 93.5% with LPS-induced TLR-4 activation (Fig. 1G). To assess potential intracellular stores, collaborators analyzed semi-permeabilized TLR4-activated neutrophils via 2% paraformaldehyde (PFA). Flow cytometry analysis detected fourfold higher fluorescence intensity levels of DEspR+ expression but same % of DEspR+CD11b+ neutrophils in PFA-semi-permeabilized neutrophils (Fig. 1H), compared to non-PFA-permeabilized TLR4-activated neutrophils (Fig. 1G). This observation suggests intracellular stores of DEspR, in addition to cell surface DEspR, hence higher “total” DEspR+ expression intensity, concordant with cell membrane, cytoplasmic and nuclear DEspR+ expression detected by immunocytology (Fig. 1A, Supplementary Fig. S1A).

Relevant to ex vivo analysis, these observations indicate that EDTA-anticoagulated blood exhibit less susceptibility to ex vivo experimental changes with increases in time and temperature (Fig. 1E, Supplementary Fig. S1C). For quantitative ex vivo ARDS patient sample analysis, we therefore used only EDTA anti-coagulated whole blood processed within 1-h from sampling from hereon, in order to minimize confounders that increase DEspR-expression ex vivo. This will avoid overestimating actual circulating levels in patient samples and false positives.

To further confirm DEspR+ expression in neutrophils, we analyzed the requisite co-expression of DEspR and adenosine deaminase acting on RNA (ADAR1) RNA-editase, since DEspR protein-translation is RNA-editase dependent^18^. Using flow cytometry analysis of PFA-fixed, Triton-X permeabilized human neutrophils, we show that every DEspR+ neutrophil is ADAR1+ (Fig. 1I–K), as seen in DEspR+ Panc1 tumor cells^18^. Co-expression in neutrophils exhibits 0.51 to 0.74 Pearson correlation coefficient, p < 0.0001 (Fig. 1J,K), thus supporting ADAR1+ neutrophils as a marker corroborating DEspR+ human neutrophils.

### Detection of DEspR+ neutrophils in ARDS and COVID-19-ARDS lung tissue-sections

**Figure 2:**
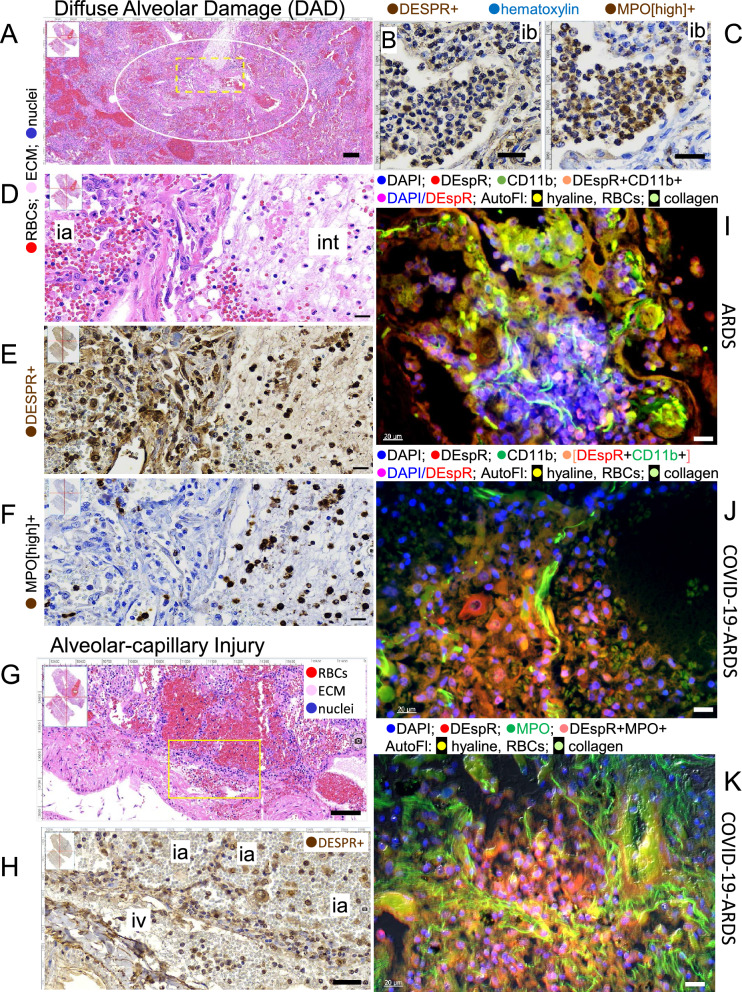
DEspR+ neutrophils and monocytes in ARDS post-mortem lung sections. (**A**). H&E section showing area (white ○) of diffuse alveolar damage (DAD) in post-mortem lung section from ARDS patient. Yellow dashed □, region of Interest (ROI) analyzed in higher magnification in panels D-I). (**B**) Immunohistochemical-diaminobenzidene (IHC-DAB)-staining of bronchiolar exudate (ib, intrabronchial) and transmural infiltrates showing DEspR+ expression, and (**C**) myeloperoxidase (MPO)+ expression, hematoxylin as counterstain. (**D**) Higher magnification of Region of Interest (ROI) with diffuse alveolar damage (DAD) shown in (A). (**E**) IHC-DAB staining of adjacent serial sections showing DEspR+ expression in inflammatory cells in DAD-ROI, (F) serial section to panel-E showing MPO+ [high-expression] IHC-DAB staining typically seen in neutrophils. The 5X lower levels of MPO+ expression in macrophages were not detected in conditions used; hematoxylin counterstain. (**G**) Representative H&E image of area with acute alveolar injury with intra-alveolar hemorrhages in lung sections also exhibiting diffuse alveolar damage in another area. Yellow box depicts ROI shown in (**H**). (**H**) Representative IHC-DAB staining for DEspR+ expression in area of acute alveolar injury. DEspR+ neutrophils and monocytes in the intravascular lumen (iv), parenchyma, alveolar walls and intra-alveolar (ia) spaces. (**I**) Representative DEspR and CD11b double IF-staining of post-mortem lung tissue-section from ARDS patient; DEspR+CD11b+ neutrophils and macrophages in the intra-alveolar spaces and lung parenchyma; autofluorescence from red blood cells (RBCs), hyaline membranes, and collagen fibrils. (**J**) Representative DEspR and CD11b IF-staining of COVID-19-ARDS lung tissue section: DEspR+CD11b+ neutrophils and macrophages in the intra-alveolar spaces and lung parenchyma; autofluorescence from red blood cells (RBCs), hyaline membranes, and collagen fibrils. (**K**) Representative DEspR and MPO IF-staining of COVID-19-ARDS lung tissue section: DEspR+MPO+ neutrophils and monocye/macrophages in intra-alveolar spaces; autofluorescence of hyaline membranes, collagen fibrils. Bar = 20 microns. Supplementary Fig. S2 for single fluorophore panels for IF-staining I-K.

To determine whether DEspR+ neutrophils are present in ARDS patient lung tissue, we performed immunohistochemistry analyses of post-mortem serial lung-tissue sections from patients with ARDS (n = 8) in regions of diffuse alveolar damage (Fig. 2A–I) as well as, in areas of alveolar-capillary injury (Fig. 2J–K). Using an anti-DEspR mouse-recombinant mAb of hu6g8, hu6g8-m, immunohistochemistry with DAB chromogen (IHC-DAB) was optimized in order to track spatial patterns of DEspR+ expression in lung tissue-sections. This detected DEspR+ neutrophils in intrabronchiolar exudate, along with some DEspR(−) neutrophils identified by the characteristic polylobulated nuclei (Fig. 2B).

In adjacent serial sections, IHC-DAB immunostaining conditions limited to detection of only high myeloperoxidase (MPO[high])+ expression typical in neutrophils with 5 × higher expression than monocytes^31,32^,detected primarily MPO[high]+ neutrophils in the intrabronchiolar exudate (Fig. 2C). In areas of diffuse alveolar damage (DAD) (Fig. 2A,D–F), IHC-DAB analyses of serial sections detected intra-alveolar, intravascular and intraparenchymal DEspR+ neutrophils and monocytes (Fig. 2E) and MPO[high]+ neutrophils (Fig. 2F). Similarly, in other lung areas with acute alveolar-capillary injury changes (Fig. 2G), we also detected DEspR+ neutrophils and monocytes in the intravascular, parenchymal and intra-alveolar spaces (Fig. 2H). Confirmatory IF-staining detected intra-alveolar and interstitial DEspR+CD11b+ neutrophils and macrophages in ARDS (Fig. 2I, Supplementary Fig. S2A) and COVID-19-ARDS (Fig. 2J, Supplementary Fig. S2B) lung tissue sections, as well as DEspR+MPO+ neutrophils and macrophages in COVID-19-ARDS lung tissue section (Fig. 2K, Supplementary Fig. S2C). These observations validate measuring peripheral levels of DEspR+CD11b+ neutrophils and monocytes by flow cytometry in ARDS and COVID-19-ARDS patient whole blood samples.

### Analysis of DEspR-pathway gene-network in COVID-19-neutrophils

Since DEspR’s ADAR1 RNA-edited transcript^18^ is not discernable in scRNA-sequencing limited to 300 nucleotides from the poly-A sequence of each transcript to ascertain specificity and equivalent representation, we studied the DEspR-pathway gene network (DEspR’s modulators, ligands, and downstream function-marker) represented in the scRNA-seq data files of immune cells and epithelial cells in nasopharyngeal and broncho-lavage fluid samples from COVID-19 subjects^12^: healthy control (n = 5), mild (n = 8) and severe (n = 11) COVID-19 patient samples. Based on the significant correlation of intracellular DEspR+and ADAR1+ human neutrophils detected by flow cytometry (Fig. 2J–K), we assigned ADAR1+ neutrophils in scRNA-seq data files as an estimate-indicator of the maximum level of DEspR+ neutrophils.

**Figure 3:**
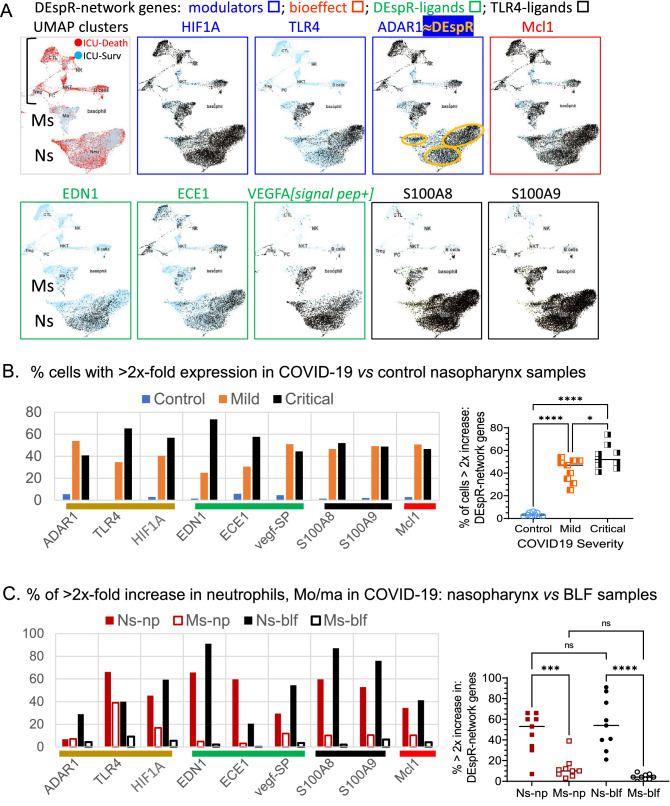
Single cell RNA-seq profiles of genes that modulate DEspR expression: DEspR-expression network. (**A**) Profiles of scRNA-seq analysis showing UMAP cluster depicting neutrophils (Ns), monocytes (Ms) and lymphocytes (Ls) identified by scRNA-sequence profiles of broncholavage fluid samples from critically ill COVID-19-ARDS patients on ventilator (n = 2), and corresponding scRNA-seq levels of DEspR-modulators: HIF1A, modulator of DEspR transcription, at RNA level (HIF1A stabilized in normoxia by activated TLR4), RNA-editing of DEspR transcript (ADAR1 RNA-editase), mobilization to cell surface upon activation (TLR4), DEspR-bioeffect marker for pro-survival role (Mcl1), DEspR-ligands ET1 (EDN1, ECE1), and VEGFA-signal peptide (propeptide VEGF), and TLR4-activators during cell injury – alarmins: S100A8 and S100A9. Yellow open circle, encircles estimate (≈) DEspR+ neutrophils based on flow cytometry findings that every DEspR+ neutrophil is ADAR1+. DEspR-RNA edited mRNA is beyond scRNA-seq 300 nt-seq from poly-A tail. No expression (blue circle), ≥ 2 × expression (filled circle). (**B**) Quantitative scRNA-seq database analysis of DEspR-expression network genes measured as % of cells in sample with ≥ 2x-fold increase in RNA levels in nasopharyngeal samples comparing non-COVID-19 control patients (n = 5, mean ± sd: 3.1 ± 1.9), mild COVID-19 patients (n = 8: 42.6 ± 10.2), and critically ill COVID-19 patients (n = 11: 54.1 ± 10.6). Non-parametric one-way ANOVA with Tukey’s multiple comparisons testing: *, p = 0.0226, ****, p < 0.0001. (**C**) Quantitative scRNA-seq database analysis of DEspR-expression network genes comparing % ≥ 2 × expression for each gene among neutrophils (Ns) and monocytes-macrophages (Ms) in nasopharyngeal (np) and broncholavage fluid (blf) samples, (n = 9 genes in DEspR-expNetwork per cell type) in COVID-19-patient samples. Average % expression of all 9 genes in Ns-np: (mean ± sd: 46.78% ± 19.9); Ms-np: (12.7% ± 10.7); Ns-blf: (55.3% ± 25.1), and Ms-blf (4.2% ± 2.8). Non-parametric one-way ANOVA with Sidak’s multiple comparisons testing: ***, p = 0.0006; ****, p < 0.0001.

Comparative scRNA-seq analysis showed that positive modulators of DEspR transcription (TLR4 and hypoxia inducible factor 1-alpha, HIF1A), DEspR RNA-editing for translation (ADAR1 RNA-editase), DEspR cell-surface mobilization (TLR4), TLR4-endogenous activators (alarmins S100A8/S100A9) and DEspR downstream-function marker (Mcl1), are all predominantly expressed in neutrophils, along with DEspR’s two ligands, ET1 (EDN1), and the signal peptide in VEGFA-propeptide (vegf-SP) (Fig. 3A). Interestingly, neutrophil expression of the endothelin converting enzyme (ECE1), required for release of ET1 from its pro-peptide (Fig. 3A) suggests neutrophil self-sufficiency to produce mature ET1, and hence, a putative ET1/DEspR autocrine loop. In contrast, transcripts for other ET1 receptors: ETA-receptor (ETAR) and ETB-receptor (ETBR), VEGF-A receptor: (KDR), and the other S100A8/A9 alarmins receptor: (AGER) were minimally, if not at all, detected in neutrophil scRNA-sequences (Supplementary Fig. S3A–D).

Furthermore, expression levels of all 9 genes representing DEspR’s expression and functional network are significantly increased in COVID-19 compared to healthy controls (Fig. 3B), and in neutrophils compared to monocytes-macrophages in nasopharyngeal and broncho-lavage COVID-19 samples (Fig. 3C). The basis to study neutrophils is further supported by scRNA-seq documentation of increased expression of receptors to cytokines increased in ARDS^33^ and/or in COVID-19-ARDS^34^, such as: interleukin (IL)-IL-6, IL-8, IL-1β, IL-18, and tissue necrotic factor-alpha (TNF-α)^35^ (Supplementary Fig. S3G). These observations provide molecular evidence linking neutrophils as effectors of “cytokine storms” in ARDS, concordant with the known association of increased neutrophils with delayed apoptosis and mortality in ARDS^1,36,37^ and COVID19-ARDS^38^.

### Detection of DEspR+ CD11b+ neutrophil-subset in ARDS and COVID-19-ARDS

To further study the DEspR+ neutrophil-subset, we completed a prospective pilot observational study of consented patients diagnosed with ARDS based on the Berlin Definition before the COVID19 pandemic. We prospectively studied consented ARDS patients (pre-COVID pandemic) regardless of the underlying acute disease trigger and associated comorbidities (Supplementary Table S1 for demographics).

**Figure 4:**
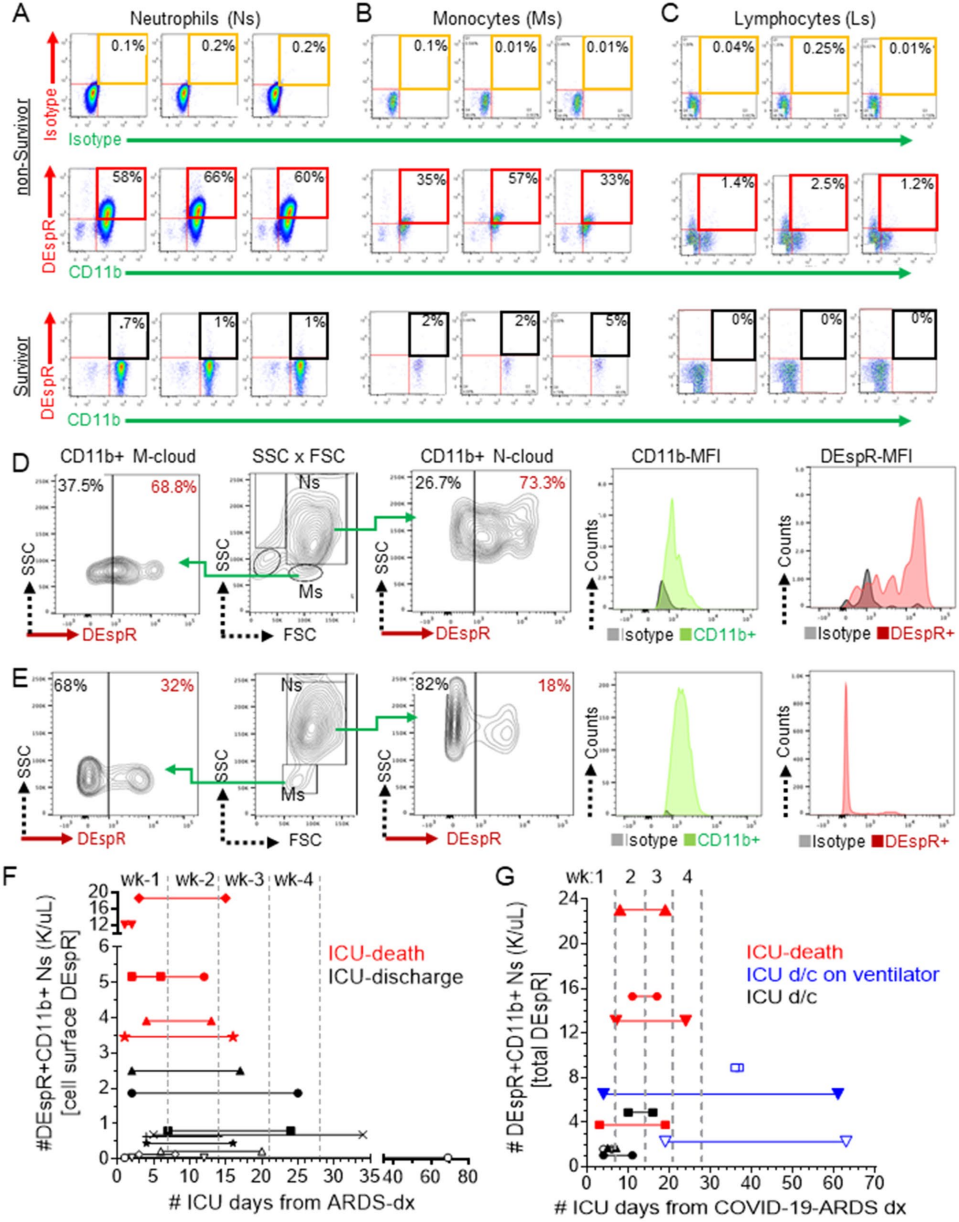
Analysis of DEspR+ neutrophils in ARDS and COVID-19-ARDS patients. (**A–C**) Representative FCM analysis, done in triplicates, of neutrophils (Ns), monocytes (Ms) and lymphocytes (Ls) in non-survivor vs survivor patient with ARDS. Corresponding isotype controls *vs* double immunotyping with anti-DEspR (hu6g8) and anti-CD11b. Quadrant 2 (Q2) for DEspR+CD11b+ neutrophils, monocytes and/or lymphocytes. (**D–E**) Representative FCM-analysis of PFA-fixed samples from patient with COVID-19-ARDS, mechanically ventilated, 61 days in the ICU (**D**) compared to (**E**) COVID-19-ARDS patient discharged after 6 days in the ICU. CD11b+DEspR+ neutrophils (Ns) (contour plot and histogram of fluorescence intensities), and monocytes (Ms). (**F–G**) Graph of duration of ICU-stay (days) from day of FCM-analysis of DEspR+CD11b+ Ns (1st symbol) until discharge or death (2nd symbol), stratified by level of number (#) of cell surface DEspR+CD11b+ neutrophils (K/μL) detected. Time zero marks day of ARDS diagnosis in non-COVID-19 ARDS (F), and in COVID-19-ARDS (G). d/c, discharge; wk, week.

First, we ascertained DEspR-specific immunotyping of whole blood samples from ARDS patients by validating our gating strategy for flow cytometry (Supplementary Fig. S4A–E), DEspR-specific detection in duplicates (Supplementary Fig. S4F–I) and reproducibility in triplicates (Fig. 4A, Supplementary Fig. S4J). We note that the level of DEspR+CD11b+ neutrophil counts was not simply due to age in ARDS and COVID-19-ARDS (Supplementary Fig. S4K,L respectively).

With this ascertainment, we then prospectively studied 19 ARDS patients (pre-COVID-19 pandemic), then 11 COVID-19-ARDS patients in the ICU at Boston Medical Center, by FCM-analysis. To assess for putative differences in ARDS pre-COVID-19 pandemic, we compared extremes in the clinical spectrum: a non-survivor with ARDS-MOF compared with an ARDS survivor discharged from the ICU in 4 days. FCM-analysis of cell-surface DEspR+ expression showed increased levels of DEspR on CD11b+ activated neutrophils (Fig. 4A) and monocytes (Fig. 4B), and on CD11b[-] lymphocytes (Fig. 4C) in ARDS-nonSurvivor, in contrast to minimal DEspR+ expression in the ARDS-survivor (Fig. 4A–C). Fluorescence intensity histograms corroborate DEspR-specific immunotyping and differential expression in triplicates (Supplementary Fig. S4J). With experimental ascertainment of reproducibility of DEspR-specific immunotyping, from hereon, studies were done in duplicates.

In COVID19-ARDS patients, we prospectively studied 11-subjects (Supplementary Table S1 for demographics). Mandated by institutional safety requirements, we studied disinfected (4% paraformaldehyde or PFA) whole blood samples from COVID-19-ARDS patients, and performed FCM analysis within 1 h from blood draw. FCM-analysis of subjects representing extremes of the clinical severity spectrum also detected increased total number DEspR+ neutrophils and monocytes in a patient with severe COVID-19-ARDS requiring 61 days intensive care unit (ICU)-care (Fig. 4D), compared with a patient with milder COVID-19-ARDS discharged after 6 days in the ICU (Fig. 4E).

Observing differential levels at the polar ends of the clinical spectrum of severity, we next stratified mortality outcomes in ARDS (Fig. 4F) and COVID-19-ARDS (Fig. 4G) patients by levels of DEspR+CD11b+ neutrophil-counts (K/μL whole blood). These pilot study trend-maps show an emerging differential pattern between survivors and non-survivors in ARDS and COVID-19-ARDS, providing bases for correlation analyses.

### Association of DEspR+ CD11b+ neutrophil-subset with ARDS severity and mortality

**Figure 5:**
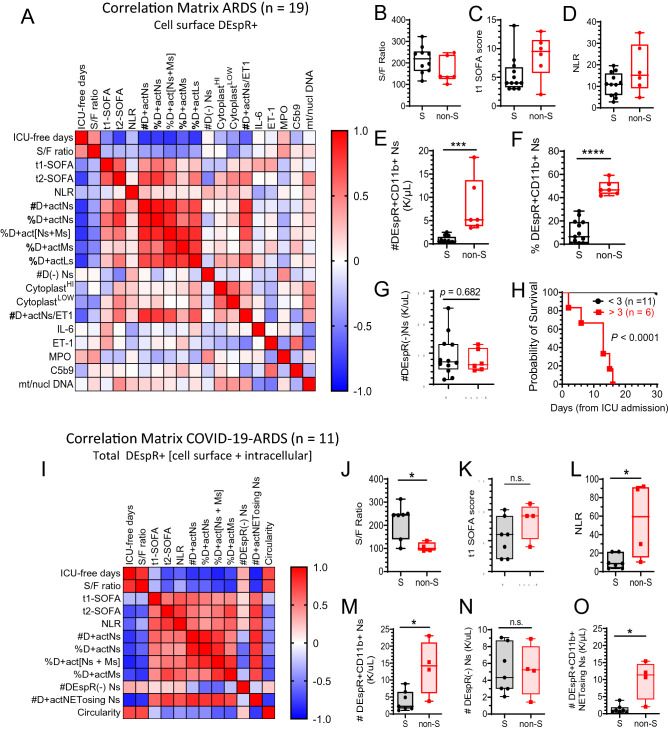
Correlation matrix analysis of DEspR+ neutrophils, clinical parameters and plasma biomarkers in ARDS and COVID-19-ARDS. (**A**) Correlation matrix ARDS: Spearman Rank Correlation, n = 19 patients with ARDS diagnosis (Berlin definition) of cell surface DEspR+ expression levels (% or number #) in CD11b+ activated neutrophils (actNs), monocytes (actMs), and lymphocytes (actLs); number #DEspR[-] neutrophil-counts; and their corresponding correlation with 1] clinical parameters of severity [ICU-free days at 28 days, defined as (28 minus # of ICU-days) with ICU-death = [− 1], and > 28 days in the ICU = 0; SpO2/FiO2 or S/F ratio, Sequential Organ Failure Assessment (SOFA) scores on day of FCM-analysis (t1-SOFA), SOFA on day of ICU-discharge or ICU-death (t2-SOFA)]; 2] plasma biomarkers reported to be elevated in ARDS by others [interleukin-6 (IL-6), endothelin-1 (ET1), myeloperoxidase (MPO), terminal complex of complement (sC5b9), and ratio of number of copies of mitochondrial/nuclear DNA (mt/nucl DNA), and 3] flow cytometry parameter linked to NET-formation (cytoplast levels with high or low granularity (SSC). See Table 1 for specific values: correlation coefficient *rho*, P-values with power > 0.8. Graphs of ARDS survivors (S, n = 13), non-survivors (non-S, n = 6): (**B**) S/F ratio as indicator of hypoxemia, survivors (mean ± sd: 214.7 ± 62.1), non-survivors (164.9 ± 60.8), p value not significant; (**C**) SOFA score, survivors (mean ± sd: 5.4 ± 3.1) and non-survivors (8.7 ± 3.8); p value not significant; (**D**) NLR: survivors (mean ± sd: 10.96 ± 5.4), non-survivors (18.03 ± 11.21); p value not significant. (**E**) number (#) of cell-surface DEspR+CD11b+ neutrophils (Ns): survivors (mean ± sd: 0.8035 ± 0.8), non-survivors: (8.1 ± 6.0); two-tailed Mann Whitney p = 0.0001 (***), effect size Hedges’ g less 4% correction: 2.03. (**F**) % of DEspR+CD11b+ neutrophils in ARDS: survivors (mean ± sd:10.3 ± 10.0), non-survivors (48.2 ± 6.3); two-tailed Mann Whitney p < 0.0001 (****), effect size Hedge’s g with 4% correction: 4.03. (**G**) number of cell surface DEspR[-] neutrophils (Ns): survivors (mean ± sd: 9.19 ± 6.7), non-survivors: (7.67 ± 3.5); two-tailed Mann Whitney p = n.s. (**H**) Kaplan-Meir Survival curve analysis with threshold for DEspR+CD11b+ neutrophil-counts set at 3 K/μL whole blood as determined from Fig. 5E; Log rank (Mantel-Cox) test Chi square 20.56, *P* < 0.0001, Hazard Ratio (Mantel–Haenszel) 90.5, 95% CI of ratio: 12.91 to 634.7. (**I**) Correlation matrix COVID-19-ARDS: n = 11 (severe COVID-19 requiring mechanical ventilation). Parameters as defined in (**A**), except for #D+ actNs which represents total (intracellular + cell surface) DEspR+ CD11b+ neutrophils detected, and addition of number (#) of DEspR+CD11b+ NET-forming neutrophils (D+ actNET-forming Ns), and circularity index of neutrophils (low circularity < 0.8 indicative of NET-forming neutrophil, see Methods). Analysis of COVID-19-ARDS survivors (S, n = 7) vs non-survivors (non-S, n = 4) in the following parameters. (**J**) S/F ratio, survivors (mean ± sd: 219.1 ± 73.3), non-survivors (105.6 ± 19.9), Mann Whitney p = 0.024. (**K**) t1-SOFA scores (on day of FCM analysis) survivors (mean ± sd: 5.6 ± 3.2), non-survivors (8.25 ± 3.0), Mann Whitney test not significant. (**L**) NLR: survivors: (mean ± sd: 10.7 ± 7.7), non-survivors: (55.3 ± 41.4), two-tailed Mann Whitney p = 0.0242 (*), effect size Hedge’s g with 4% correction: 1.73. (**M**) Number (#) DEspR+ CD11b+ neutrophil-counts (K/μL) in whole blood: survivors (mean ± sd: 3.8 ± 2.99), non-survivors: (13.8 ± 7.9), two-tailed Mann Whitney p = 0.04 (*), effect size Hedge’s g with 4% correction: 1.82. (**N**) Number (#) of cell surface DEspR[-] neutrophils: survivors (mean ± sd: 5.22 ± 2.8), non-survivors: (5.20 ± 3.1); two-tailed Mann Whitney p = n.s. (**O**) #DEspR+ CD11b+ NET-forming neutrophil-counts (K/μL) whole blood: survivors: (mean ± sd: 1.3 ± 1.29), and non-Survivors: (10.0 ± 5.7), two-tailed Mann Whitney p = 0.0121 (*), effect size Hedge’s g with 4% correction: 2.4.

To dissect the differential pattern emerging between survivors and non-survivors (Fig. 4F,G), we first performed correlation matrix analysis on a panel of DEspR-based flow cytometry markers, clinical markers of ARDS severity, and plasma biomarkers associated with neutrophil-mediated secondary tissue injury, and ET1 one of two DEspR ligands (Fig. 5A, Table 1). To assess clinical severity, we studied the number of ICU-free days at day 28 from ARDS diagnosis as a measure of mortality (death scored as [-1]) and speed to recovery within 28-days^39^, ARDS severity (SpO2/FiO2 or S/F ratio measure of hypoxemia), and Sequential Organ Failure Assessment (SOFA) scores on the day of sampling for flow cytometry analysis (t1-SOFA) and on day before ICU-discharge or ICU-death (t2-SOFA). To assess context in ARDS pathogenesis, we studied biomarkers of pathogenic events in ARDS relevant to neutrophil-mediated secondary tissue injury: interleukin-6 (IL-6 marker of cytokine storms), soluble C5b9 (terminal complex of complement activation), myeloperoxidase or MPO (neutrophil activation), ratio of the number of copies of mitochondrial to nuclear DNA in plasma (mtDNA-NET-formation)^24^, and DEspR+CD11b+ cytoplasts, (anuclear remnants of neutrophils associated with “vital NETosis”)^25^.

**Table 1:** Spearman rank correlation matrix analysis: ARDS.

BioMarkers	Clinical measures of Severity in ARDS-multiorgan failure (MOF)							
	ICU-free days (d28)		S/F ratio		t1-SOFA		t2-SOFA	
	*r*	*p*-value	*R*	*p*-value	*r*	*p*-value	*r*	*p*-value
**CBC-differential**								
NLR	− 0.27	0.296	− 0.24	0.368	− 0.12	0.638	0.08	0.778
**Flow cytometry**								
#DEspR + CD11b + Ns	*− 0.80*	*0.0002*	− 0.38	0.152	0.42	0.095	*0.71*	*0.003*
%DEspR + CD11b + Ns	*− 0.78*	*0.0001*	− 0.41	0.119	**0.56**	**0.016**	*0.79*	*0.0003*
%DEspR + CD11b + [Ns + Ms]	*− 0.81*	*0.0003*	− 0.51	0.052	0.44	0.090	**0.54**	**0.042**
%DEspR + CD11b + Ms	*− 0.64*	*0.009*	− 0.48	0.075	0.12	0.647	0.19	0.488
%DEspR + CD11b + Ls	*− 0.79*	*0.0003*	− 0.36	0.171	0.24	0.354	**0.52**	**0.039**
#DEspR(−) Ns	0.04	0.871	0.03	0.906	− 0.24	0.362	− 0.49	0.054
Cytoplast^HI^	− 0.29	0.254	0.01	0.960	− 0.24	0.344	0.27	0.312
Cytoplast^LOW^	− 0.39	0.120	− 0.44	0.098	0.13	0.610	0.47	0.067
**Plasma biomarkers**								
#DEspR + CD11b + Ns/ET1	*− 0.63*	*0.014*	− 0.23	0.427	0.19	0.512	**0.55**	**0.045**
IL-6	0.02	0.927	− 0.05	0.859	0.35	0.157	− 0.01	0.986
ET1	− 0.07	0.801	0.08	0.797	0.35	0.194	0.03	0.923
MPO	0.28	0.253	0.39	0.132	− 0.23	0.356	− 0.25	0.325
sC5b9	− 0.29	0.243	0.10	0.706	0.08	0.763	0.06	0.824
mt/nucl DNA	− 0.04	0.898	0.15	0.616	− 0.06	0.810	0.44	0.099

n = 19 subjects ARDS, all cause [pre-COVID19 pandemic].

*CBC-differential* NLR, neutrophil lymphocyte ratio calculated from ratio of absolute neutrophil to absolute lymphocyte counts.

*Flow cytometry parameters* #DEspR+ CD11b+ Ns, total number (#) in K/μL of DEspR+ C11b + neutrophils (Ns); %DEspR + CD11b + Ns, % of DEspR + CD11b + neutrophils among total (CD11b+/−) neutrophils; %DEspR + CD11b + [Ns + Ms], sum of the % of DEspR + CD11b + neutrophils and monocytes; %DEspR + CD11b + Ms, %DEspR + CD11b + monocytes among total (CD11b+/−) monocytes; %DEspR+CD11b+ Ls, %DEspR+CD11b+ lymphocytes among total (CD11b+/−) lymphocytes; #DEspR(−) Ns, total number (#) in K/µL of DEspR(−) neutrophils (Ns). cytoplast^HI^, cytoplasts with high SSC or granularity; cytoplast^LO^, low SSC or low granularity cytoplast;

*Plasma biomarkers* Plasma levels of ET1, endothelin-1, IL-6, interleukin-6; MPO, myeloperoxidase levels; sC5b9, soluble complement terminal C5b9-complex; and mt/nucl DNA, ratio of mitochondrial DNA copy number to nuclear DNA copy number.

*Clinical measures of ARDS severity* ICU-free days by day 28 = [28 minus # ICU days] with NonSurvivors = [− 1] and Survivors > 28 ICU-days = 0; S/F ratio, SpO2/FiO2 ratio as a measure of hypoxemia severity; SOFA, **S**equential **O**rgan **F**ailure **A**ssessment score; t1-SOFA, SOFA score on day of flow cytometry analysis; t2-SOFA, SOFA score at end of ICU stay.

*Statistical analysis* Spearman Rank Order Correlation coefficient *rho* (*r)* effect size: strong *r* 0.6–0.79; very strong *r* 0.8–1.0. Data points are peak values for subjects with multiple FCM analyses. Spearman Rank Correlation Coefficient (*rho, r* > 0.61, p < 0.05, has Power > 0.8 with n = 19; significant Spearman *rho* with power 0.8 highlighted in italics; significant Spearman *rho* but power < 0.8 in bold.

Spearman rank correlation matrix analysis detected significant negative correlation between the number of DEspR+CD11b+ activated neutrophils and ICU-free days at day 28 (Fig. 5A, Table 1). Other DEspR-based FCM-parameters, such as % of DEspR+CD11b+ neutrophils, monocytes and lymphocytes, also showed significant negative correlation with ICU-free days at day 28 (Table 1), in contrast with DEspR[-] neutrophil-counts which exhibited no significant correlation (Table 1). Spearman correlation analysis also detected significant correlation of number (#) and per cent (%) of DEspR+CD11b+ neutrophil with t2-SOFA scores but not with t1-SOFA (Fig. 5A, Table 1), supporting a pathogenic role in MOF-progression. In contrast, DEspR[-] neutrophil-counts, NLR, plasma levels of IL-6, MPO, and sC5b9 did not correlate with any of the measures studied (Fig. 5A, Table 1).

Next, we analyzed differences in group medians between ARDS-patient survivors and non-survivors (Fig. 5B–G). In this pilot study, we found no significant difference between survivors and non-survivors for clinical measures of ARDS severity: S/F ratio (Fig. 5B), t1-SOFA scores (Fig. 5C), and prognostic measure NLR (Fig. 5D). In contrast, significant difference in medians (Mann Whitney p = 0.0001) with large effect size (Hedge’s g > 0.8) was detected for DEspR+CD11b+ neutrophil-counts (#) (Fig. 5E) and % DEspR+CD11b+ neutrophils (Fig. 5F), but not for DEspR[-] neutrophil-counts (Fig. 5G). Kaplan Meier survival curve analysis with a threshold for DEspR+CD11b+ neutrophil-counts set at 3,000/μL (3 K/μL) whole blood showed significant differences in survival (P < 0.0001) (Fig. 5H).

### Association of DEspR+ CD11b+ neutrophils with COVID-19-ARDS severity and mortality

Similarly, in COVID-19-ARDS pilot group, Spearman rank correlation matrix analysis showed significant, strong, negative correlation of DEspR+CD11b+ neutrophil-counts with ICU-free days at day 28 from ARDS diagnosis, and with ARDS severity S/F ratio, in contrast to no correlation with DEspR[-] neutrophil-counts (Fig. 5I, Table 2). Interestingly, the sum of %DEspR+ [monocytes and neutrophils] correlated with ICU-free days at day 28 with higher Spearman correlation coefficient, significance and power than either alone (Table 2). This observation is concordant with neutrophil-monocyte intravascular-interactions reported to contribute to systemic tissue injury in acute glomerular injury^40^. In contrast, the neutrophil lymphocyte ratio (NLR) showed significant albeit less robust correlation with ICU-free days at day 28 in COVID-19-ARDs compared to DEspR+CD11b+ neutrophil-counts.

**Table 2:** Spearman Rank Correlation Coefficients: COVID-19-ARDS subjects requiring ventilator support.

Biomarkers	Clinical measures of ARDS severity							
	ICU-free days (d28)		S/F ratio		t1-SOFA		t2-SOFA	
	*r*	*p*-value	*R*	*p*-value	*r*	*p*-value	*r*	*p*-value
**CBC-differential**								
Neutrophil–lymphocyte ratio	**− 0.62**	**0.047**	− 0.34	0.313	**0.65**	**0.034**	*0.79*	*0.005*
**Flow cytometry (FCM)**								
#DEspR + CD11b + Ns	*− 0.80*	*0.004*	**− 0.63**	**0.044**	0.61	0.053	0.54	0.092
%DEspR + CD11b + Ns	*− 0.77*	*0.008*	− 0.61	0.052	0.52	0.106	0.42	0.196
%DEspR + CD11b + [Ns + Ms]	*− 0.87*	*0.0009*	**− 0.75**	**0.010**	0.43	0.193	0.54	0.092
%DEspR + CD11b + Ms	**− 0.73**	**0.014**	*− 0.80*	*0.005*	0.45	0.166	**0.74**	**0.012**
#DEspR(-) Ns	0.33	0.320	0.26	0.435	0.14	0.673	0.18	0.587
**Immunofluorescence cytology**								
#DEspR + CD11b + NET-forming Ns	*− 0.89*	*0.001*	*− 0.81*	*0.004*	0.60	0.057	*0.78*	*0.007*
Circularity index	**0.71**	**0.017**	**0.71**	**0.018**	− 0.23	0.501	− 0.52	0.102

n = 11 subjects with COVID-19-ARDS needing ventilatory support.

*CBC-differential* NLR, neutrophil lymphocyte ratio.

*Flow cytometry parameters* #DEspR+ CD11b+ Ns, total number (#) in K/μL of DEspR+C11b+ neutrophils (Ns); %DEspR+CD11b+Ns, % of DEspR+CD11b+ neutrophils among total (CD11b+/−) neutrophils; %DEspR+ CD11b+ [Ns+Ms], sum of the % of DEspR+ CD11b+ neutrophils and monocytes; %DEspR+CD11b+ Ms, %DEspR+CD11b+ monocytes among total (CD11b+/−) monocytes; #DEspR(-) Ns, total number (#) in K/µL of DEspR(−) neutrophils (Ns).

*Immunofluorescence Cytology parameters* #DEspR+CD11b+ NET-forming Ns: number of DEspR+CD11b+ NET-forming Ns (% DEspR+ CD11b+NET-forming Ns x total number of DEspR+CD11b+ Ns); Circularity index, circularity index as a quantitative measure of NET-forming neutrophils with extruded DNA resulting in irregular perimeters with decreased similarity to a circle: outline of DAPI+ DNA fluorescence closest to ‘perfect circle’ = 1. Threshold 0.8: a NET-forming neutrophil has < 0.8 circularity index.

*Clinical measures of ARDS severity* ICU-free days by day 28 = [28 minus # ICU days] with nonSurvivors = [− 1] and Survivors > 28 ICU-days = 0; S/F ratio, SpO2 converted to PaO2/FiO2 ratio as a measure of hypoxemia severity; SOFA, Sequential Organ Failure Assessment score; t1-SOFA, SOFA score on day of flow cytometry analysis; t2-SOFA, SOFA score at end of ICU stay (or day prior to death or discharge).

*Statistical analysis* Spearman Rank Order Correlation coefficient *rho* (*r*) effect size: strong *r* 0.6–0.79; very strong *r* 0.8–1.0. Data points are peak values for subjects with multiple FCM analyses. Spearman Correlation Coefficient *r* > 0.76, alpha < 0.05, Power > 0.8, n = 11 (italics); Spearman *r* > 0.6, alpha < 0.05, power 0.7 to 0.8 (bold).

Comparative analysis of COVID-19-ARDS survivors and non-survivors showed significant differences in medians for S/F ratio (Fig. 5J) but not for t1-SOFA score (Fig. 5K). Concordant with correlations detected, significant differences and large effect size were also detected between survivors and non-survivors for NLR (Fig. 5L), DEspR+CD11b+ neutrophil-counts (Fig. 5M), but not for DEspR[-] neutrophils (Fig. 5N).

To compare our pilot study observations with emerging biomarkers of severe COVID-19, we performed a retrospective analysis of COVID-19-ARDS patients requiring ventilatory support at Boston Medical Center. Data corroborate significant differences in NLR (Supplementary Fig. S5A–D), concordant with reports that increased NLR is an independent predictor of mortality in ARDS and COVID-19^41^. In contrast, C-reactive protein did not show significant differences between survivors and non-survivors (Supplementary Fig. S5E–H).

### Characterization of DEspR+ NET-forming neutrophils in ARDS and COVID19-ARDS

**Figure 6:**
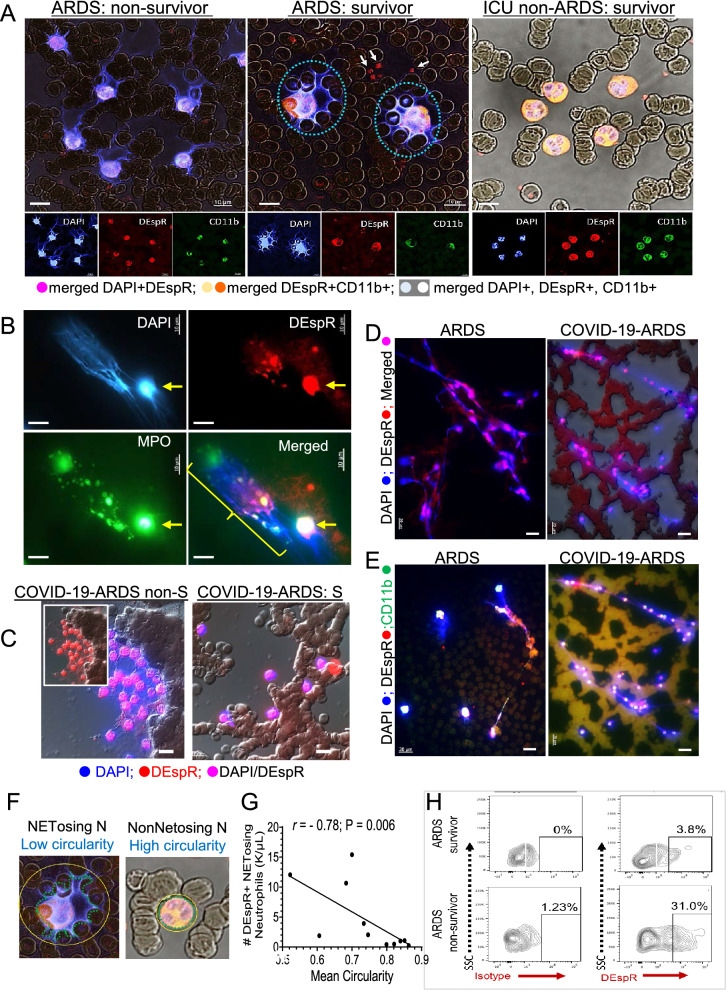
DEspR+ NET-forming neutrophils in ARDS and COVID-19-ARDS. **(A**) Representative epifluorescence DIC (differential interference contrast) images of IF-stained blood smears from ARDS-non-survivor (left), ARDS-survivor (middle), and non-ARDS critically ill (right) patient in the ICU. DEspR+ CD11b+ NET-forming neutrophils with extruded DAPI+ DNA are detected in ARDS patients, red blood cells (RBCs) in background. Arrows point to DEspR+CD11b+ microvesicles. Bar = 10 μm. (**B**) Representative image of IF-stained patient blood smear showing different NET-forming neutrophil morphologies: NET-structure with MPO+DEspR+ DNA-cloud and strands, with MPO+ or DEspR+ or MPO+ DEspR+ microvesicles within the NET. Bar = 10 μm. → , DEspR+MPO+ NET-forming neutrophil with smaller amount of DNA extruded, MPO still localized to within cell structure, short DNA-strands extruded. (**C**) Representative epifluorescence/DIC images of IF-stained blood smears from COVID-19-ARDS non-survivor and survivor. DAPI+ DEspR+ NET-forming neutrophils with extruded DNA-interconnections within multi-neutrophil clusters. Representative COVID-19-ARDS-survivor showing no multi-neutrophil clusters. RBCs in background differential interference contrast (DIC)-relief image Bar = 10 μm. (**D-E**) Representative IF-stained images of filamentous DAPI+DEspR+ extruded DNA strands with DEspR+ microvesicles (**D**), and DEspR+ CD11b+ DNA-strands with DEspR+ CD11b+ microvesicles (**E**) detected in blood smears from ARDS and COVID-19-ARDS patients. RBCs in the background. Bar = 20 μm. (**F**) Perimeter delineated demarcating irregular border of extruded DNA (DNA aqua dashed line) around a NET-forming neutrophil and surrounding adjacent RBCs from representative ARDS patient (left, identical to Panel A-middle) IF-stained blood smear showing low degree of “similarity to a circle” or circularity in NET-forming neutrophils, in contrast to high circularity of DEspR+CD11b+ non-NET-forming neutrophil (right, identical to Panel-**A** right). (**G**) Spearman rank order correlation analysis of # DEspR+CD11b+ NET-forming neutrophil-counts and mean circularity index (average from n > 500 neutrophils per patient) in COVID-19-ARDS patients (n = 11): negative correlation (r = − 0.78, P = 0.006, power > 0.8). (**H**) Representative FCM analysis of CD11b+ gated cytoplasts showing DEspR+ co-expression and granularity (SSC), comparing levels in ARDS survivor (3.8%) vs ARDS non-survivor (29.77%).

To assess NET-forming neutrophils relevant to detection of increased levels of NETs in in severe COVID-19^10,11,42,43^, we performed immunofluorescence staining to directly visualize and quantify NET-forming neutrophils in whole blood smears prepared from COVID-19-ARDS patients within 1-h from blood draw. Using high-resolution confocal imaging of immunofluorescent-staining for DEspR+CD11b+ expression, we detected differential levels of DEspR+CD11b+ NET-forming neutrophils in ARDS non-survivor, compared with ARDS-survivor and ICU-patient non-ARDS survivor (Fig. 6A).

To further characterize NET-forming neutrophils, we immunostained for MPO-positivity, a hallmark of NETs. IF-staining of blood smears detected MPO+DNA and, interestingly, DEspR+DNA in NET-forming neutrophils with the classical cytolytic NET-formation (suicidal NETosis) phenotype (Fig. 6B). Both MPO+ or DEspR+ or dual MPO+/DEspR+ micro-vesicles are detected in the DAPI+DNA-cloud and DNA-strands (Fig. 6B). In the same image, a non-cytolytic NET-forming neutrophil with extruded DNA but no vesicles and a nucleus that is DEspR+MPO+DAPI+ (Fig. 6B). We observed clustering of NET-forming neutrophils in COVID-19-ARDS non-survivors compared with COVID-19-ARDS survivors (Fig. 6C). We also observed interconnecting filamentous-DNA networks (Fig. 6D) with DEspR+ CD11b+ subcellular ‘beads’ along the DNA-strands in both ARDS and COVID-19-ARDS patient blood smears (Fig. 6E, Supplementary Fig. S6A,B).

### Quantitation and correlation analysis of DEspR+ CD11b+ NET-forming neutrophils

In order to quantify DEspR+CD11b+ NET-forming neutrophils (Ns), we used shape analysis (Fig. 6F). Semi-quantitative confocal microscopy distinguished NET-forming neutrophils with low circularity index, from non-NET-forming neutrophils with expected high circularity of 1.0 (see Supplementary Methods). Quantitative analyses of COVID-19-ARDS patient samples spanning hundreds of neutrophils per slide showed significant strong negative correlation of #DEspR+NET-forming neutrophils with mean circularity index per patient (Spearman *rho* = 0.78, p = 0.006, power > 0.8) (Fig. 6G). With this correlation, we used a circularity index < 0.8 to identify NET-forming neutrophils for quantitative analyses.

In COVID-19-ARDS patients, the total number of DEspR+CD11b+ NET-forming neutrophils correlated strongly with three clinical measures: 1] outcome at day-28 (ICU-free days at day 28), 2] degree of hypoxemia (SpO2/FiO2 or S/F ratio), and 3] severity of multi-organ failure score at end of ICU-stay (t2-SOFA), but not with t1-SOFA (Table 2, Fig. 5I). Concordantly, analysis of number of DEspR+CD11b+ NET-forming neutrophils detected significant differences (p < 0.05) in medians between COVID-19-ARDS survivors and non-survivors (Fig. 5O). In contrast, the average or peak D-dimer levels, indicative of thrombosis risk, did not exhibit significant differences between COVID-19-ARDS survivors and non-survivors (Supplementary Fig. S5I–L). Additionally, scRNA-seq profile for PADI4 linked to “suicidal NETosis” shows low (1.4%) number of neutrophils expressing PADI4 > 2X fold in COVID-19-ARDS neutrophils (Supplementary Fig. S3E).

### Analysis of DEspR+ CD11b+ and DEspR+ CD66b+ NET-remnant cytoplasts and neutrophils

Having detected DEspR+CD11b+ NET-forming neutrophils and DEspR+ MPO+ NETs on immunostained whole blood smears (Fig. 6A,B), we analyzed levels of cytoplasts as circulating NET-remnants by flow cytometry. We and another research site detected elevated DEspR+CD11b+ cytoplast levels in ARDS subjects (Fig. 6H) however, we did not observe association between circulating DEspR+CD11b+ cytoplast levels with clinical measures of ARDS severity (Fig. 5A, Table 1). Nevertheless, an independent small pilot study of patients with sepsis, and sepsis-ARDS confirmed elevated DEspR+CD66+ cytoplasts^44^ and neutrophils in contrast to low to no levels in healthy donors (Supplementary Fig. S6C–H). As a marker for neutrophil-degranulation, CD66b confirms neutrophil-derived cytoplasts. We note that neutrophils were isolated from whole blood samples via inertial microfluidic separation from RBCs^45^, performed however, ~ 3-h from blood draw—hence accounting for the much higher levels > 90% DEspR+ cytoplasts and neutrophils observed.

### Ex vivo analysis of DEspR-inhibition in ARDS-patient neutrophils

**Figure 7:**
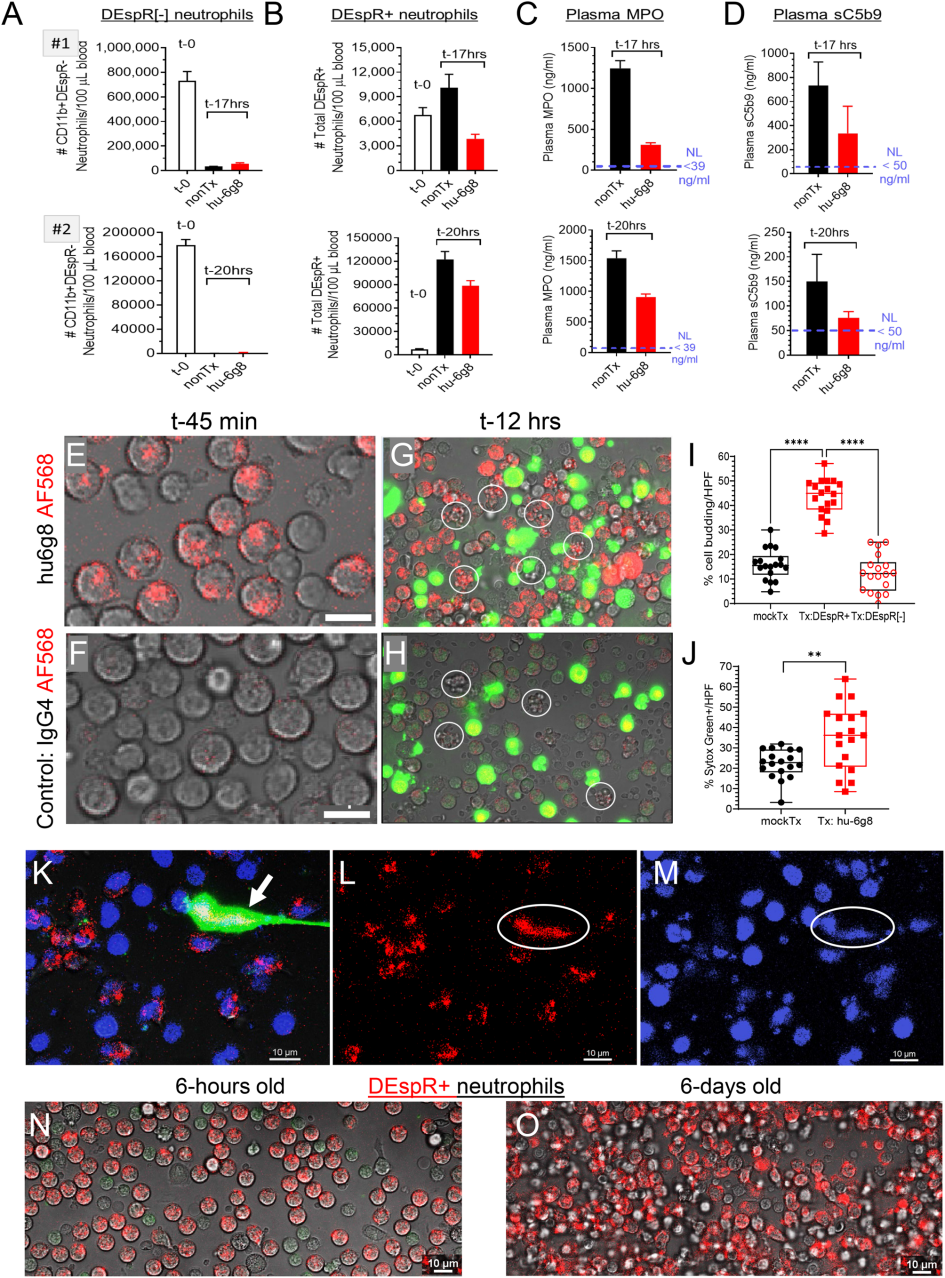
Effects of DEspR-inhibition on neutrophil survival: ex vivo analysis of ARDS-patient and non-human primate (*Rhesus macaque*) neutrophils. (**A–D**) Analysis of DEspR[-] and DEspR+ neutrophils obtained from two patients with ARDS at baseline (< 1 h from sampling), and after incubation with/without hu6g8 treatment (100 μg/ml) at 37 °C with simulated shear for 17 h (patient #1), or 20 h (patient#2). Flow cytometry assessed number of (**A**) DEspR[-] vs (**B**) DEspR+ surviving neutrophils compared to baseline. ELISA analyses of (**C**) MPO and (**D**) soluble terminal complex of complement (sC5b9) after ex vivo hu6g8 treatment. Normal MPO and sC5b9 levels notated. (**E**) Representative image showing hu6g8 target engagement and internalization into *Rhesus* DEspR+ neutrophils at t-45 min from onset of exposure to AF568-labeled hu6g8. (**F**) AF568-labeled IgG4 isotype control showing no target engagement or internalization into *Rhesus* DEspR+ neutrophils. (**G**) Representative image at t-12 h from video-recorded live cell imaging of isolated white blood cells (WBCs) documented to have > 90% DEspR+ CD11b+neutrophils among all neutrophils , and exposed to 10 μg/ml hu6g8-AF568 labeled antibody at 4 °C × 20 min to eliminate non-specific cell uptake by macropinocytosis or endocytosis. DEspR+ (red circle) *Rhesus*-neutrophils, apoptotic cell budding (encircled ○), and Sytox Green (SytoxG)-positive membrane permeable (). (**H**) Representative t-12 h image of isotype hu-IgG4 AF568 shows minimal to no isotype-AF568 (red circle) uptake; constitutive apoptosis cell budding (encircled ○), SytoxGreen-positive staining in cells with loss of cell membrane integrity (). (**I**) Quantitation of *Rhesus* neutrophils (from 2 *Rhesus* donors: donor-1, 6 HPFs/chip, donor-2, 12 HPFs in 2 independent chips) exhibiting apoptotic-typical cell budding. Analysis of n = 18 HPFs each for IgG4-isotype mock-treated control and hu6g8-treated groups distinguish DEspR(+) and DEspR(−) neutrophils, 15–56 cells/HPF, % apoptotic cell budding: (mean, 95% CI of means) mockTx (16%, 13–19%), Tx: DEspR+ (44%, 40–47%), Tx:DEspR[-] (12%, 8.6–16%). One way ANOVA with Tukey’s multiple comparisons test: p < 0.0001 (****). (**J**) Quantitation of SytoxG+ non-viable cells per high power field (n = 18 HPFs/in IgG4-isotype mock-treated vs hu6g8-treated group, > 16–50 cells/HPF), mean, 95% CI of mean for mockTx isotype control (22.2, 18.6–25.7), for hu6g8Tx (35.2, 27.3–43.2); two tailed t-test p = 0.0033 (**). (**K**) Photomicrograph image from live cell imaging of cytolytic NET-forming (“suicidal NETosing”) neutrophil (→) detected ~ t-15 min after addition of SytoxGreen: DEspR-AF568 (red), nucBlue+ DNA (blue), Sytox-Green detects externalized DNA; merged yellowish-white (DEspR+ red, SytoxGreen+, nucBlue +). (**L**) hu6g8-AF568 bound to or internalized in DEspR+ neutrophils (red); (**M**) nucBlue+ DNA (blue). (**N**) *Rhesus* neutrophils analyzed on day of blood draw: confocal photomicrograph obtained at t-1 h of live-cell imaging shows internalized hu6g8-AF568 in DEspR+ neutrophils. (**O**) Rhesus neutrophils analyzed 6 days from blood draw: live-cell imaging confocal photomicrograph at t-1 h from onset of hu6g8-AF568 binding detects DEspR+ neutrophils.

To determine bioeffects of DEspR-inhibition, we analyzed ARDS patient whole blood with humanized anti-DEspR IgG4^S228P^ antibody, hu6g8, added as ex vivo treatment for 17–20 h overnight with rotation to prevent aggregation. Controls comprised of patient-specific mock-treated and baseline pre-treatment controls (Fig. 7A). Comparative FCM-analysis showed that compared to baseline levels and after 17–20 h of ex vivo incubation at 37 °C, DEspR+ neutrophils increased in number compared with markedly decreased number of DEspR[-] neutrophils (Fig. 7A,B), suggesting that normal neutrophilic constitutive apoptosis is delayed in DEspR+ neutrophils but not in DEspR[−] neutrophils, and that some DEspR[−] neutrophils became DEspR+ with time.

To determine DEspR’s role in delayed apoptosis, we inhibited DEspR via hu6g8-treatment, and performed flow cytometry after 17–20 h of treatment ex vivo. This showed that hu6g8 decreased the number of DEspR+ neutrophils in ARDS patient whole blood (Fig. 7B). Hu6g8 also reduced myeloperoxidase (MPO) (Fig. 7C) and soluble terminal complex of complement (sC5b9) (Fig. 7D) plasma levels, in contrast to greater than twofold increased levels in mock-treated controls, respectively after 17–20 h ex vivo incubation. These observations indicate that hu6g8 induced neutrophil apoptosis and function-shutdown of neutrophil-complement system reciprocal co-activation after 17–20 h of DEspR-inhibition via hu6g8-treatment. Importantly, neutrophil scRNA-seq profile for CD47, the "don’t eat me signal” is minimal, with only 0.3–0.91% of neutrophils with > 2 × fold CD47 (n = 19 COVID-19 patients) (Supplementary Fig. S3F). This supports the therapeutic hypothesis that induction of apoptosis in DEspR+ neutrophils with no CD47 “don’t eat me signal” can be cleared by efferocytosis.

### Ex vivo analysis of DEspR-inhibition in Rhesus macaque neutrophils

To further test that DEspR-inhibition induces apoptosis in DEspR+ CD11b+ neutrophils, we performed live cell imaging of *Rhesus macaque* neutrophils exposed to fluorescently labeled hu6g8-AF568 or fluorescently labeled human IgG4-AF568 isotype control for 20 min at 4 °C to avoid non-specific endocytosis. We selected *Rhesus* neutrophils as model system since *Rhesus*-to-human neutrophils are more similar than human-to-mouse neutrophils^46^.

We first validated the presence of circulating DEspR+ CD11b+ neutrophils in *Rhesus* via flow cytometry using identical conditions to ex vivo analysis of ARDS patient samples (Supplementary Fig. S7A–H). Next, we studied hu6g8 target engagement, internalization, and induction of characteristic apoptosis cell-budding bioeffects by confocal live cell imaging of *Rhesus* neutrophils. We exposed *Rhesus* neutrophils to either AF568-labeled anti-DEspR hu6g8 antibody (treatment) or IgG4-isotype (mock-treatment) control for 20 min at 4 °C to avoid non-specific endocytosis. After removing excess unbound antibody, 24-h live cell imaging was initiated with video-recordings. At t-45 min, live-cell images detected target engagement and internalization of hu6g8-AF568 antibody (Fig. 7E, Supplementary Fig. S7I) but not in the isotype control (Fig. 7F). Specificities were confirmed throughout with representative t-12 h timepoint images (Fig. 7G,H). At t-12 h, live cell imaging showed more apoptotic cell budding changes in NHP-neutrophils with internalized hu6g8 (Fig. 7G). In the isotype control, apoptotic cell budding was detected concordant with neutrophil constitutive apoptosis (Fig. 7H). SytoxGreen impermeable dye uptake marked loss of cell viability. Both cell death indicators increased with time (Fig. 7G,H).

At the 12-h midpoint, quantitation of apoptotic cell changes and SytoxGreen-positive non-viability were done. Quantitative analysis of 18 high power fields (HPFs) with 20–50 cells/HPF representing three independent experimental fields of view showed that hu6g8 induced apoptosis in DEspR+ neutrophils significantly greater than levels seen in isotype-treated control NHP cells (Fig. 7I). Importantly, hu6g8 induced apoptosis greater than constitutive apoptosis occurring in DEspR[-] cells unaffected by hu6g8 treatment (Fig. 7I). Interestingly, loss-of-viability staining by Sytox Green occurred in neutrophils not undergoing apoptotic cell budding, and was also slightly greater in hu6g8-treated neutrophils compared with isotype mock-Tx controls (Fig. 7J), indicating that DEspR-inhibition may facilitate other programmed cell-death in neutrophils via decreased CIAP2 as observed in anti-DEspR mAb-treated pancreatic cancer stem cells^47^.

Interestingly, shortly after the addition of SytoxGreen at t-15 min of live cell imaging, NET-formation with cytolysis was observed with DEspR+nucBlue+SytoxGreen+ NETs (Fig. 7K–M). DEspR+ neutrophils with internalized AF568-labeled hu6g8 were also observed (Fig. 7K). This contrasts t-12 h (Fig. 7G) where no NET-formation events were observed up to 24 h, suggesting the hypothesis that anti-DEspR induces neutrophil-apoptosis and pre-empts NET-formation. Lastly, NHP neutrophils shown to be > 90% DEspR+ at baseline flow cytometry analysis and day-1 live cell imaging (Fig. 7N) exhibited marked delayed apoptosis as live DEspR+ neutrophils were still observed 6-days after blood sampling (Fig. 7O).

## Discussion

Data from multiple experimental systems among multi-center collaborators testing neutrophils in whole blood samples, either from NHVs, ARDS or COVID-19-ARDS patients, or *Rhesus macaques*, reproducibly identify the DEspR+ CD11b+ neutrophil-subset, concordant with cumulative evidence for neutrophil heterogeneity^1,48^. Detection of DEspR+ intracellular stores and increase in DEspR+CD11b+ neutrophils upon LPS-TLR4 activation in NHV blood samples indicate a dynamic subset-response to TLR4-activation. Importantly, the identification of the DEspR+CD11b+ neutrophil-subset in whole blood is supported by detection of DEspR+CD11b+ and DEspR+MPO+ neutrophils and monocyte/macrophages in postmortem lung tissue sections from ARDS and COVID-19-ARDS. Detection in the lung interstitium, intra-alveolar spaces, and vascular lumen in association with either diffuse alveolar damage (DAD) or alveolar-capillary injury confirm identification of the DEspR+ neutrophil-subset.

In marked contrast to DEspR[-] neutrophils, the subset-specific correlations of DEspR+CD11b+ neutrophil-counts with severity and mortality measures in both ARDS and COVID-19-ARDS suggests pathophysiological relevance of DEspR+CD11b+ neutrophils as a neutrophil-subset. Additionally, the characteristics of DEspR+CD11b+ neutrophils observed in ex vivo analyses: delayed apoptosis, a predisposition to intravascular NET-formation and neutrophil-NET clustering—further support DEspR+CD11b+ neutrophils as a pathophysiological ‘rogue’ neutrophil-subset. Notably, these characteristics of DEspR+CC11b+ neutrophils are concordant with observations of neutrophil changes in ARDS and COVID19-ARDS patients^1,11,30,36^.

Consistent with expectations of a ‘rogue’ neutrophil-subset implicated in progression of ARDS and COVID-19-ARDS, increased RNA levels of the DEspR-pathway gene network detected in neutrophil-specific COVID-19-ARDS scRNA-seq data files interconnect in multiple positive autocrine loops. These autocrine loops span not just ligand-receptor levels, (DEspR, ET1, VEGF-sp), but also transcriptional modulators of DEspR itself, its ligands and required RNA-editase (HIF1A and TLR4), and DEspR’s modulator for cell-surface expression (TLR4) and TLR4’s endogenous ligands (S100A8/A9). These autocrine loops provide a potential self-sustaining framework that would be expected of a neutrophil-subset implicated in progressive MOF. These observations are concordant with elevated TLR4^49^, S100A8/A9^14,49,50^, ADAR1^51^, and ET1^52^ detected in clinical (ARDS, or COVID19-ARDS) and pre-clinical model studies of ARDS, COVID-19-ARDS.

The direct visualization of DEspR+CD11b+ neutrophils with extruded DNA and intact nucleus and cell membrane in ARDS and COVID-19-ARDS patient whole blood smears match characteristics of NET-formation with mitochondrial DNA (mtDNA) and intact cell membranes^24,53^, and scanning electron microscopy images of NET-forming neutrophils^54^. Direct visualization of NET-forming neutrophils in patient blood smears documents intravascular events at time of blood draw, delineates NET-forming neutrophils as source of extruded DNA, and gives insight into pathophysiological context of different NET-subtypes. The algorithm-based automated quantitation of NET-forming neutrophils provides an objective quantitative method for direct morphological identification of NET-forming neutrophils in fixed patient blood smears, thus overcoming the limitation of non-quantitative visualization of IF-stained NETs^55^. These observations are supported by markedly elevated ratio of plasma mitochondrial DNA (mtDNA) to nuclear DNA (nDNA) copy-number in ARDS samples. Since elevated mtDNA released by necrotic cells is observed in ARDS^56^ and COVID-19-ARDS^57^, using mtDNA/nDNA ratio could help further dissect mtDNA-NET levels from cell necrosis which would exhibit a mtDNA/nDNA ratio < 1 given that mtDNA is less than 1% of nuclear DNA.

Detection of the classic DEspR+MPO+ DNA-cloud, DEspR+CD11b+ NET-forming neutrophils with intact cell membranes, and long DNA-remnants with DEspR+CD11b+ microvesicles on the DNA strands in the same patient blood smear altogether indicate ongoing multiple intravascular NET-formation events in ARDS and COVID-19-ARDS. Multiple NET-subtypes are placed into pathophysiological context and are not due to confounders from different methods of analysis, sample source or procurement^53,58–60^. Relative pathophysiological relevance is supported by the significant correlation of intravascular DEspR+/CD11b+ NET-forming neutrophil-counts with multiple clinical measures of severity in COVID-19-ARDS, in contrast to non-correlation of NLR, IL-6, and sC5b9^61,62^ in this prospective observational study.

Additionally, the presence of DEspR+CD11b+ ~ 200 micron-long intravascular DNA-strands, multi-NET-forming neutrophil clusters, and NET-DNA strands straddling RBCs, are concordant with prior reports in COVID-19-ARDS and ARDS^1,10,11,60^. This combination of multiple intravascular NET-structures likely cause intravascular flow impedance leading to low-flow ischemia with or without micro-thromboses. Intravascular impedance to flow without microthrombi can account for persistence of low-flow or micro-ischemic events in different organs in severe ARDS and COVID-19-ARDS despite pharmacological thromboprophylaxis or anti-thrombotic treatment^63^.

Data showing that DEspR-inhibition leads to neutrophil apoptosis in ARDS patient and *Rhesus macaque* samples support DEspR as an actionable therapeutic target. Induction of apoptosis in dysregulated, apoptosis-resistant neutrophil-subset(s)^64^ implicated in progressive secondary tissue injury leading to ARDS-MOF^5^ has been deduced to be a critical step towards initiation of resolution of excessive inflammation in ARDS^1^. Hence, targeted-inhibition of DEspR+ neutrophils with endpoint induction of neutrophil apoptosis presents a potential therapeutic approach with advantages. First, hu6g8-mediated induction of DEspR+ neutrophil apoptosis attains function-shutdown of neutrophil-complement reciprocal co-activation^65^, hence potentially breaking this self-sustaining pro-cell injury mechanism. Second, induction of DEspR+ neutrophil apoptosis without cell lysis provides a key step for efferocytosis^66^ of the DEspR+ neutrophil subset. Thirdly, induction of apoptosis in DEspR+ neutrophils preempts progression to NET-formation, hence a potential therapeutic approach to preventing DEspR+ dysregulated NET-formation. This gains relevance when neutrophils are PADI4-negative, as seen in 98.6% of neutrophils in the COVID-19-ARDS RNA-seq files, hence non-responders to PADI4-inhibitors of NET-formation. While more studies are needed to elucidate mechanisms, induction of DEspR+ neutrophil apoptosis by DEspR inhibition complies with prior delineation that stopping neutrophil-mediated tissue injury requires induction of neutrophil apoptosis^1,64^.

Importantly, consideration for potential on-target side effects, especially in the context of acute kidney injury as part of multi-organ failure in ARDS, highlights known DEspR+ expression in human medullary tubular epithelial cells^67^. In the presence of immunoglobinuria, anti-DEspR antibody passing through the glomerulus could present potential on-target tubular epithelial effects, but unlikely as antibody functionality will be altered in the increasingly acidic and hyperosmotic milieu in the kidney medullary lumen. Equally important, the critical limitation of pleiotropic neutrophil inhibitors in the context of ongoing infections and risk for secondary infections as in ARDS and COVID-19-ARDS, the target-specific inhibition of DEspR+ rogue neutrophils will spare DEspR[-]CD11b+ activated neutrophil subsets, hence preserve neutrophil defense functions against infections pertinent to critically ill patients with ARDS or COVID-19-ARDS.

Altogether, data identify the DEspR+CD11b+ neutrophil-subset as a therapeutic target with the potential to break the feed-forward progression of neutrophil-mediated tissue injury in ARDS and COVID-19-ARDS, while preserving DEspR[-] neutrophil functions. Data provide foundational basis for further study.

### Limitations

We acknowledge the limitations of prospective pilot observational studies with n = 19 ARDS and n = 11 COVID-19-ARDS, and n = 19 COVID-19 scRNA profiles. With a focus on the study of neutrophils, we did not evaluate other cells with cytotoxic capabilities. We acknowledge the inherent limitations of the study of critically ill patients with limited patient samples for study, and limitations in our COVID-19-ARDS whole blood samples treated with 4% PFA to inactivate SARS CoV2 virus [final 2% PFA], leading to non-availability of plasma samples to perform ELISA studies on biomarkers and MPO-DNA complexes of NET-remnants.

## Materials and methods

### Study design

Different tasks in this interdisciplinary pilot observational study among different collaborators were compartmentalized in order to attain blinding of researchers during task-performance. The following tasks were compartmentalized: [a] patient screening, [b] consenting and blood sampling, [c] processing of blood for flow cytometry and FlowJo analysis, [d] clinical data collection, [e] laboratory testing—ELISAs; [f] preparation of blood smears from whole blood; [g] immune-fluorescence staining, [h] confocal microscopy imaging and semi-quantitative measures; [i] analysis of collated laboratory and clinical data. The diagnosis of ARDS was determined in real time by review of ICU diagnoses, and checked by clinician collaborators post-hoc blinded to all experimental results from flow cytometry, immune-fluorescence staining, and ELISA results.

### Study subjects

All subjects were identified in the ICU under study protocols approved by the Institutional Review Board (IRB) of Boston University (IRB H-36744). Each subject’s legal authorized representative gave written informed consent for study participation in compliance with the Declaration of Helsinki.

We enrolled 19 ARDS patients in the pre-COVID-19 pandemic period, and 11 COVID-19 ARDS patients admitted to the intensive care unit (ICU) at Boston Medical Center. ARDS diagnosis was based on clinical diagnosis using the Berlin Definition. COVID-19 ARDS patients were ascertained as COVID-19 positive by SARS-CoV-2 PCR testing. Additional data were obtained prospectively from 16 COVID-19 ARDS patients to examine the time-course during ICU-hospitalization and correlation of other known markers with survival: neutrophil lymphocyte ratio, C-Reactive Protein and D-Dimer. Collaborators enrolled NHVs (MM), patients with severe COVID-19 in the ICU for bronchial-lavage fluid studies (RE), healthy donors (n = 2) and patients with sepsis-ARDS (n = 4) for inertial microfluidic separation (BDL, RMB, MPV) according to respective institutional guidelines.

### Blood collection

Fresh normal peripheral blood neutrophils were procured from AllCells, LLC (CA), which were shipped overnight in Iscove’s Modified Dulbeco’s Medium (IMDM) with 2 mM EDTA, and 0.5% Bovine Serum Albumin (BSA), 4 °C.

For study of ARDS and COVID-19-ARDS patient samples, whole blood (3 or 6 mls) was collected via pre-existing indwelling peripheral vascular lines into K2-EDTA vacutainer tubes (FisherScientific, MA) from patients hospitalized in the ICU at Boston Medical Center by the ICU-nurse. COVID-19 patient EDTA-anticoagulated blood samples were immediately fixed with one volume of 4% PFA. Both Non-COVID and COVID-19 blood samples were processed for flow cytometry analysis within 1 h from blood collection. Platelet poor plasma was isolated and frozen at − 80 °C for future testing within 2 h from blood draw. Blood smears were prepared within 1 h from blood draw.

*Flow cytometry analysis of blood samples* [See Supplementary Methods for details.]

At BUSM, EDTA-anticoagulated blood samples from non-COVID ARDS subjects (100 μl per tube, × 2–3 replicates) were processed for flow cytometry within 1-h from blood sampling. [See Supplementary Methods for detail] Flow cytometry buffer comprised of Hank’s balanced salt solution plus 2% heat-inactivated FBS as blocking agent; staining antibodies: 10 μg/ml of AF-647 labeled hu6g8 mAb, or the corresponding human IgG4-AF647 isotype IgG4, and 2.5 μg/ml anti-CD11b-AF488 or the corresponding mouse IgG1 kappa isotype control, AF-488; staining done at 4 °C × 30 min with rotation and protected from light; after staining, cells were fixed in 1% PBS-buffered PFA pH 7.4 at 4 °C, followed by RBC lysis at RT. After final wash, stained cells were resuspended in 400 μl HBSS 2% FBS, filtered and analyzed on a BD LSR-II flow cytometer. Analysis was done using FloJo Flow Cytometry Analysis Software (www.FloJo.com). Controls used were: both fluorescence minus one (FMO) controls, both isotype controls, compensation beads for both staining antibodies to check labeled antibody quality.

For disinfected COVID-19 blood samples (2%PFA-fixed), samples were washed 3 times with 8 volumes of HBSS + 2% FBS to remove residual fixative prior to processing for flow cytometry as described above. Each test sample run in duplicates.

At BWH, EDTA-anticoagulated whole blood samples were processed 2–3 h from sampling and white blood cells were separated from RBCs via Inertial Microfluidic Separation validated previously for neutrophil characterization^44^. Flow cytometry was performed immunotyping for CD45, CD66b and DEspR at room temperature × 20 min, and analyzed on an LSR-Forteza for low FSC/SSC cytoplasts. [See Supplementary Methods for Details.]

### Western blot analysis

Western blot analysis was done essentially as described^47^ using equal amounts of total cellular protein extract (25 μg) isolated from human neutrophils. Neutrophil cell extracts were prepared by cell homogenization in 3 volumes of 1 × Laemmli buffer (Bio-Rad). Human kidney protein extract was used as control. Proteins were size-separated on a 15% Tris–HCL SDS-PAGE (Bio-Rad) and transferred to PVDF membrane (Bio-Rad). The Western blot was reacted with anti-DEspR antibody (hu6g8) at 20 μg/ml for 18 h at 4 °C with shaking. Immunoreactive proteins were detected by chemiluminescence using the ECL Western Detection kit (Thermo Scientific 34077).

### Immunohistochemistry of tissue sections

We analyzed postmortem human lung sections from patients with clinical diagnosis of ARDS, and pathological diagnosis of diffuse alveolar damage (DAD). Immunohistochemistry was performed at Horus Scientific, Inc using DAB (3,3’-diaminobenzidine) and hematoxylin counter stain. Chimeric anti-DEspR hu6g8 with mouse IgG2a backbone was used at 1:100 dilution (~ 10 μg/ml), and anti-human myeloperoxidase antibody 1:50 dilution. Primary antibodies were incubated for 16 h at 4 °C. Negative controls were run without primary antibodies, positive controls were run using DEspR+ xenograft tumor sections^47^.

### scRNA-Seq database analysis

scRNA-Seq data of two patients with critical COVID-19 disease courses (WHO stage 4), covering nasopharynx, protected specimen brush swabs of the airways, and bronchial lavage fluid were obtained from the UCSC Cell Browser generated by studies performed at Charité—Universitätsmedizin Berlin and Berlin Institute of Health. Patient cells were processed using the 10X Chromium system with v3.1 chemistry. Primary analysis was performed using Cell Ranger 3.2.0 with a hg19 reference genome, followed by removal of ambient RNA using SoupX 1.2.2. Preprocessing and primary of analysis of the scRNA-Seq data were performed using Seurat 3.1.4. For details on patient characteristics, sample processing, and data analysis, please refer to Chua et al.^12^ Visualization of the expression of genes of interest was performed using the UCSC Cell browser and confirmed using Seurat 3.2.2 of original datasets. Expression values shown are normalized to the total count of unique molecular identifiers (UMIs) per cell.

### Ex-vivo LPS treatment of human normal volunteer (HNV) neutrophils

[See Supplementary Methods for details.]

At Fraunhofer ITEM, heparinized whole blood was stored on ice until processing and used within 1-h after collection. Whole blood (100 µl) samples were washed with 1 ml of ice cold assay buffer, and cells were incubated in 100 µl of assay buffer containing bacterial endotoxin lipopolysaccharide LPS (100 ng/ml; Escherichia coli serotype 0111:B4) or assay buffer as control for 1 h at 37 °C. The reaction was then stopped, cells washed, then resuspended and cells were stained with hu6g8-PE (10 µg/ml) and CD11b-FITC for 30 min on ice under constant stirring in the dark. Cells were washed to remove unbound antibodies, fixed for 10 min at 4 °C, followed by RBC lysis. The cell pellet was resuspended in 250 µl flow cytometry buffer and was analyzed within 2 h using a Beckman Coulter Navios 3L 10C flow cytometer and data analyzed using Beckman Coulter Kaluza 2.1 Software.

At BUSM, 100 μl EDTA-anticoagulated whole blood samples (n = 6) were exposed to 75–100 μg/ml LPS at 37 °C × 1-h, then subjected to FCM analysis as described above.

### Plasma level analysis of biomarkers by ELISA and quantitation of mitochondrial DNA

Individual ELISA protocols were performed as per manufacturer’s instructions with the following sample dilutions: For MPO ELISA (abcam cat# ab195212) plasma dilution 1:1000; for C5b9 ELISA (MyBioSource cat# MBS2021557) plasma dilution 1:100; for IL-6 ELISA (Abcam cat# ab46027) plasma dilution 1:2; for ET1 ELISA (abcam cat# ab133030) plasma dilution 1:2.

To compare the levels of mitochondrial to nuclear DNA in human plasma samples we used the NovaQUANT™ Human Mitochondrial to Nuclear DNA Ratio Kit (SIGMA-Aldrich cat# 72,620-1KIT) as per manufacturer’s instructions. The kit measures the mtDNA copy number to that of nuclear DNA by Real-Time PCR of specific mitochondrial and nuclear genes optimized for equivalent amplification. Plasma DNA was isolated from 200 μl of plasma using the Quick-cfDNA Serum & Plasma Kit (Zymo Research, cat# D4076) as per manufacturer’s instruction.

### Immunofluorescence staining of NET-forming neutrophils

Blood smears were prepared by capillary action from EDTA anticoagulated whole blood (10 μL) samples on a Superfrost Plus Microscope slide (Fisher Scientific, cat# 12-550-15) within 1-h from blood sampling. Blood smears were air dried for 10 min then fixed with 100% Methanol (chilled to − 20 °C) for 10 min. Fixed slides were stored dry in − 20 °C freezer for future immunostaining. Immunofluorescence (IF)-staining to detect NET-forming neutrophils was done as described^68^, with custom modifications. We used anti-DEspR hu6g8 and anti-CD11b, as well as anti-DEspR and anti-MPO antibodies—conjugated to fluorophores (AF568, or AF488) for direct pair-wise immunostaining; DAPI for DNA detection. Chilled methanol fixation and permeabilization allowed fixation within 1 h from blood draw, eliminating need for paraformaldehyde and saponin. PBS with 5% FBS was used as blocking and binding solution for primary antibodies.

### Fixed cell imaging of blood smears for quantiation of NET-forming neutrophils

Immunofluorescence imaging was performed as contract research service at Nikon Imaging Laboratory (Cambridge MA). Slides were imaged with a Nikon Ti2-E Widefield microscope equipped with a Plan Apo λ 20 × objective and Spectra LED light source and controlled by NIS-Elements. Briefly, an automated, JOBS routine in NIS-Elements was used to image 100 evenly spaced positions along an entire slide. At each position, focus was automatically adjusted with the Perfect Focus System (PFS) and then sequential images with the 395 (Blue), 470 (Green) and 555 (Red) nm LED light sources to detect DAPI (nuclei), Alexa Fluor 488 (CD11b) and Alexa Fluor 568 (DEspR, hu6g8), respectively. Each stack of 100 images was then processed with a General Analysis 3 algorithm in NIS-Elements to segment the nuclei, measure their circularity (Circularity = 4π [area/perimeter^2^], area of minimum circle enclosing NET-forming neutrophil, perimeter of NET-forming neutrophil with all DNA-extrusions], and quantify the signal intensity of any co-localized CD11b and DEspR expression. Data were exported to a CSV file where the final scoring is completed in Excel. [See Supplementary Methods for details.]

### Ex-vivo anti-DEspR treatment of ARDS patient blood samples

One ml of freshly obtained blood samples were incubated overnight at 37 °C with or without anti-DEspR mAb (hu6g8 at 100 μg/ml). After incubation half of the samples were subjected to FACS analysis as described above and the other half was processed for plasma isolation. Plasma MPO and C5b-9 levels were determined with corresponding ELISA kits as described above.

### Quantitation of apoptotic cell changes and viability after anti-DEspR treatment of non-human primate (NHP) DEspR+ CD11b+ neutrophils by live cell imaging

Briefly, whole blood from *Rhesus macaque* provided by Biomere (Biomere Biomedical Research Models, Inc., Worcester MA) was analyzed by flow cytometry to determine the number of DEspR+CD11b+ neutrophils. White blood cells (WBCs) were then obtained, washed and resuspended in Hank’s Balanced Salt Solution (HBSS) + 2% Fetal Bovine Serum (FBS). WBCs were counted, divided into aliquots and incubated with 10 µg/ml Alexa Fluor 568-conjugated hu6g8 antibody or Alexa Fluor 568-conjugated IgG4 isotype antibody for 20 min at 4 °C. Cells were washed to remove unbound antibody, then concentrated at to approximately 10^8^ cells/ml in RPMI, then loaded into imaging device. Live cell imaging was performed using a microfluidic chip with three parallel conjoined microfluidic channels, and a confocal microscope (Ti2-E microscope equipped with Nikon A1R HD25 point scanner and 60X Plan Apo λ Oil objective) housed within a temperature and CO_2_-controlled incubator. Images were then acquired every minute for the first 9 h, and then every 5 min for 15 h thereafter, for a total of 24 h observation time. At 15 min into imaging, Sytox Green (Thermo-Fisher) was added into the imaging media for each chip at a final concentration of 1:6000. (See Supplementary Methods for detail).

### Statistical analysis

For demographics, statistical comparisons of clinical parameters between the non-COVID and COVID-19 ARDS subjects we used the Fisher Exact test (GraphPad Prism v9.0.1) comparing corresponding proportions, except for age, S/F ratio and SOFA score which were done by using a two-tailed Mann Whitney (GraphPad Prism v9.0.1). For survivor vs non-survivor group comparisons, we used the two-tailed Mann Whitney test (GraphPad Prism v9.0.1) with effect size calculated via Hedge’s g with 4% correction. Correlations were calculated by using the Spearman Rank Order correlation test (GraphPad Prism v9.0.1) and power calculations determined by using SigmaPlot 11.0 software. All data sets conformed to the assumptions of each specific statistical test. P < 0.05 was considered statistically significant, sufficient power 0.8.

### Human subject studies

All subjects were identified in the ICU under respective human subject study protocols approved by respective Institutional Review Boards (IRB): Boston University School of Medicine IRB H-36744; Brigham and Woemen’s Hospital, Harvard Medical School IRB approval number for healthy donors 2002P000272 and sepsis patients samples 2008P000495; Ethics Committee of the Hannover Medical School, No. 839–2010. Normal human volunteers and each subject’s legal authorized representative gave written informed consent for study participation in compliance with the Declaration of Helsinki.

### Non-human primate blood sample studies

NHP blood samples were provided by Biomere Biomedical (Worcester, MA) in compliance with Biomere’s institutional guidelines. Biomere holds Animal Welfare Assurance with the Office of Laboratory Animal Welfare, NIH, PHS: assurance number A4324-01, USDA registration number 14- R-0192 and AAALAC file number 1152.

## Supplementary Information

Supplementary Information.
