# Supplementary material for: A targetable ‘rogue’ neutrophil-subset, [CD11b+DEspR+] immunotype, is associated with severity and mortality in acute respiratory distress syndrome (ARDS) and COVID-19-ARDS
Source: Sci Rep. 2022 Apr 4;12:5583. doi: 10.1038/s41598-022-09343-1 (PMC8977568; doi:10.1038/s41598-022-09343-1)
Supplement: Supplementary file 1 — Supplementary Information. [file 41598_2022_9343_MOESM1_ESM.pdf]

Supplementary Methods.

Flow cytometry analysis of blood samples - BUSM.

At BUSM, EDTA-anticoagulated blood samples from non-COVID ARDS subjects (100 µL per tube, x 2-3 replicates) were processed for flow cytometry within 1-hour from blood sampling. Briefly, test samples were washed in 1 ml ice-cold Hank's balanced salt solution (HBSS, Invitrogen, NY) plus 2% FBS (flow cytometry FCM-buffer), centrifuged, then resuspended in 200 µL of flow cytometry buffer. Staining antibodies were then added in appropriate amounts for final concentration of: 10 µg/ml of labeled hu6g8-AF647, or the corresponding human IgG4-AF647 isotype (IgG4: ATHENS Research & Technologies, cat # 16-16-090707-4M) and 2.5 µg/ml anti-CD11b-AF488 (Invitrogen, cat#11-0118-42) or the corresponding isotype (mouse IgG1 kappa isotype control, AF-488, ThermoFisher Sci. cat# 53-4714-42). Replicate samples were incubated for 30 min at 4 ºC in the dark rotating, then washed with 1 ml FCM-buffer, then resuspended in 100 µL 1X PBS, then fixed by adding 100 µL PBS-buffered 2% PFA pH7.4 (for 1% final) for 10 min on ice protected from light. Wash off fixative with 1 ml HBSS with 2% FBS, spin, then resuspend cell pellet in 2 mls of 1x lysis buffer (BioLegend RBC Lysis Buffer, cat# 42031). Incubate for 10 min at room temperature, then spin, resuspended in 400 µl HBSS with 2% FBS, filtered and analyzed on a BD LSR-II flow cytometer (BD Biosciences). Analysis was done using FloJo Flow Cytometry Analysis Software (www.FloJo.com). Controls used were: both fluorescence minus one (FMO) controls, both isotype controls, compensation beads for both staining antibodies to check labeled antibody quality.

For disinfected COVID19 blood samples (2%PFA-fixed), samples were washed 3 times with 8 volumes of HBSS + 2% FBS to remove residual fixative prior to addition of labelled antibodies for binding. Each tube had 100 µL of fixed whole blood; each sample analyzed with duplicates.

Flow Cytometry Analysis of whole blood samples - BWH

Collaborators at BWH used EDTA-anticoagulated whole blood samples which were processed 2-3 hours from sampling. White blood cells (WBCs) were separated from RBCs via Inertial Microfluidic Separation validated previously for neutrophil characterization,[45] using 50 µL whole blood. WBC samples were then incubated with 5 µL of Fc-block (Human TruStain FcX, Biolegend) for 10 min at RT. For DEspR surface staining, leukocytes were incubated for 30 min at RT with the following antibodies to human proteins with clones noted in parentheses: anti-CD45 PerCP (HI30), anti-CD66b Pacific blue (G10F5), anti-CD16 APC-Cy7 (3G8), anti-CD14 APC (63D3) (all from BioLegend), and anti-DEspR AF568 antibody (1:50 dilution or 10 µg/ml). Following staining, cells were washed with 1 mL of PBS (without Ca^{2+} and Mg^{2+}). RBCs were then lysed and fixed with 2 ml of 1:4 dilution of Lyse/fix Buffer 5X (BD Phosflow) with distilled water for 15 min at RT. After washing, cells were resuspended and analyzed using a BD LSR Forteza flow cytometer. Data were analyzed using Flowjo software version 10.1 (Tree star) gating for neutrophils via CD45+, then CD66b+, then DEspR+ fluorescence staining for both cytoplasts (low FSC, low SSC), and neutrophils (high FSC, high SSC).

Ex-vivo LPS treatment of human normal volunteer neutrophils – Fraunhofer ITEM

At Fraunhofer ITEM, heparinized whole blood was stored on ice until processing and used within one hour after collection. Whole blood (100 µL) was washed with 1 ml of ice cold assay buffer (0.1% BSA in DPBS; centrifuged 500 x g for 5 min at 4 °C) and cells were

incubated in 100 µL of assay buffer containing LPS (100 ng/ml; Escherichia coli serotype 0111:B4, L2630 Sigma Aldrich, Germany) or assay buffer as control for 1 h at 37 °C. The reaction was stopped by adding 1 ml flow cytometry buffer containing 5% fetal bovine serum (FBS) and 2 mM EDTA in DPBS. After centrifugation (500 x g for 5 min at 4 °C), cells were stained with hu6g8-PE (10 µg/ml) and CD11b-FITC (Bear1, Beckman Coulter, Germany) for 30 min on ice under constant stirring in the dark. Cells were washed with 1 ml flow cytometry buffer and fixed with 250 µL BD CellFIX (BD Bisciences, Germany) for 10 min at 4 °C as per manufacturer's instructions followed by RBC lysis (BD Lysing Solution, BD Biosciences, Germany) as per manufacturer's instructions. The cell pellet was resuspended in 250 µL flow cytometry buffer and was analyzed within 2 hours using a Beckman Coulter Navios 3L 10C flow cytometer and data analyzed using Beckman Coulter Kaluza 2.1 Software.

At BUSM, 100µl EDTA-anticoagulated whole blood was exposed to 75-100 µg/ml LPS at 37 °C x 1-hour, then subjected to FCM analysis as described above.

Fixed Cell Imaging of Blood Smears for quantitation of NET-forming neutrophils

Immunofluorescence imaging was performed as contract research service at Nikon Imaging Laboratory (Cambridge MA) as follows. Slides were imaged with a Nikon Ti2-E Widefield microscope equipped with a Plan Apo λ 20x objective and Spectra LED light source and controlled by NIS-Elements. Briefly, an automated, JOBS routine in NIS-Elements was used to image 100 evenly spaced positions along an entire slide. At each position, focus was automatically adjusted with the Perfect Focus System (PFS) and then sequential images with the 395 (Blue), 470 (Green) and 555 (Red) nm LED light sources to detect DAPI (nuclei), Alexa Fluor 488 (CD11b) and Alexa Fluor 568 (DEspR, hu6g8), respectively. Each stack of 100 images was then processed with a General Analysis 3 algorithm in NIS-Elements to segment the

nuclei, measure their circularity and quantify the signal intensity of any co-localized CD11b and DEspR expression.

Circularity was measured using the following formula applied to non-cytolytic NET-forming neutrophils:

$$\text{Circularity} = (4 * \text{Pi} * \text{Area}) / \text{perimeter}^2$$

with area of the minimum circle enclosing NET-forming neutrophil extruded DNA; and perimeter defined as the outer border tracing of DNA-extrusions from the NET-forming neutrophil.

Data were then exported to a CSV file where the final scoring is completed in Excel. Here, cells are separated into non-NET-forming (Circularity > 0.8) and NET-forming (Circularity < 0.8) neutrophil subgroups. Moreover, cells are scored for positive (or negative) CD11b and DEspR expression, based on whether the mean signal intensities of the respective fluorophore stainings for each cell are greater (or less) than a threshold value determined from the surrounding background fluorescence. Finally, Boolean operations are used to count the number of cells in each subgroup as graded by the three markers (Yes/No NET-forming, +/- CD11b, +/- DEspR). For high resolution images, confocal images were obtained using 60X-oil immersion to assess representative images of highest and lowest circularity levels.

Quantitation of apoptotic cell changes and viability after anti-DEspR treatment of non-human primate (NHP) DEspR+CD11b+ neutrophils by live cell imaging

Whole blood from *Rhesus macaque* NHP provided by Biomere (Biomere Biomedical Research Models, Inc., Worcester MA) was analyzed by flow cytometry to determine the number of DEspR+CD11b+ activated neutrophils. Enriched WBCs from *Rhesus macaque* were

obtained after RBC lysis of NHP whole blood, washed and resuspended in Hank's Balanced Salt Solution (HBSS) + 2% Fetal Bovine Serum (FBS). WBCs were counted, divided into aliquots and incubated with 10 µg/mL Alexa Fluor 568-conjugated hu6g8 (anti-DEspR) antibody or Alexa Fluor 568-conjugated IgG4 isotype antibody for 20 minutes at 4 °C. Cells were washed with 1 mL cold HBSS + 2% FBS to remove unbound antibody and then concentrated at a very high concentration (approximately 10^8 cells/mL) prior to loading into imaging device. Labeled WBCs were kept on ice to minimize endocytosis.

Concurrently, a microfluidic plate (Organoplate 3-lane 40, Cat. No. 4004-400B, Mimetas, Leiden, Netherlands) was prepared for culturing and imaging these cells. The plate consists of 40 self-contained "chips", that each contain three parallel, conjoined microfluidic channels ("lanes"). Briefly, for each chip to be loaded with cells for imaging, collagen solution was dispensed into the middle lane and polymerized into a gel according to the manufacturer's instructions. The plate was then positioned on the stage of a confocal microscope (Ti2-E microscope equipped with Nikon A1R HD25 point scanner and 60X Plan Apo λ Oil objective) housed within a temperature and CO_2-controlled incubator (OkoLabs).

With the plate already on the microscope stage, cold, antibody-bound WBCs were dispensed into either the top or bottom lane of each collagen-loaded chip according to the manufacturer's guidelines. The experimental (hu6g8-AF568) and control (IgG4-AF568) cell suspensions were loaded into separate, adjacent chips on the plate. Pre-warmed imaging media (RPMI + 1% BSA) was then added to reservoirs connected to the channels in each chip to prevent the channels from drying out. Images were then acquired every minute for the first 9 hours, and then every 5 minutes for 15 hours thereafter, for a total of 24 hours observation time. At 15 minutes into imaging, Sytox Green (Thermo-Fisher) was added into the imaging media for

each chip at a final concentration of 1:6000. Membrane-compromised cells will fluoresce upon intercalation of Sytox Green with DNA, or will detect extruded DNA. Furthermore, to minimize evaporation of media over the course of the experiment, the chips were replenished with imaging media containing Sytox Green (1:6000) after the first 3 hours of imaging. This media replenishment washed off poorly adherent or dying cells as observed on video recording.

Supplementary Table S1. Clinical characteristics of ARDS and COVID-19-ARDS cohorts.

Characteristics	***ARDS (n = 19)***	***COVID-19-ARDS (n = 11)***	***P***
Age,mean ± SD, years	48.8 ± 17.1	60.4 ± 13.6	0.065
Male (%)	84.2	72.7	0.641
SOFA score, mean ± SD	6.5 ± 3.6	6.6 ± 3.2	0.973
S/F ratio, mean ± SD	196.0 ± 64.5	177.9 ± 81.4	0.525
	%	%	Fisher's exact test
Hypertension	50.0	72.7	0.273
Diabetes	58.8	54.5	1.000
CKD	11.8	27.3	0.353
Asthma	23.5	18.2	1.000
COPD	23.5	18.2	0.353
Obesity (BMI > 30)	25.0	45.5	0.411
Heart disease	25.0	45.5	0.411

Data are percentages except for Age, SOFA score and S/F ratio.
ARDS, acute respiratory distress syndrome; CKD, chronic kidney disease; COPD, chronic obstructive pulmonary disease. SOFA, Sequential Organ Failure Assessment; S/F ratio, SaO2/FiO2 ratio;Statistical analysis: for Age, SOFA score and S/F ratio unpaired t-test (two tail). For other characteristics Fisher Exact test.

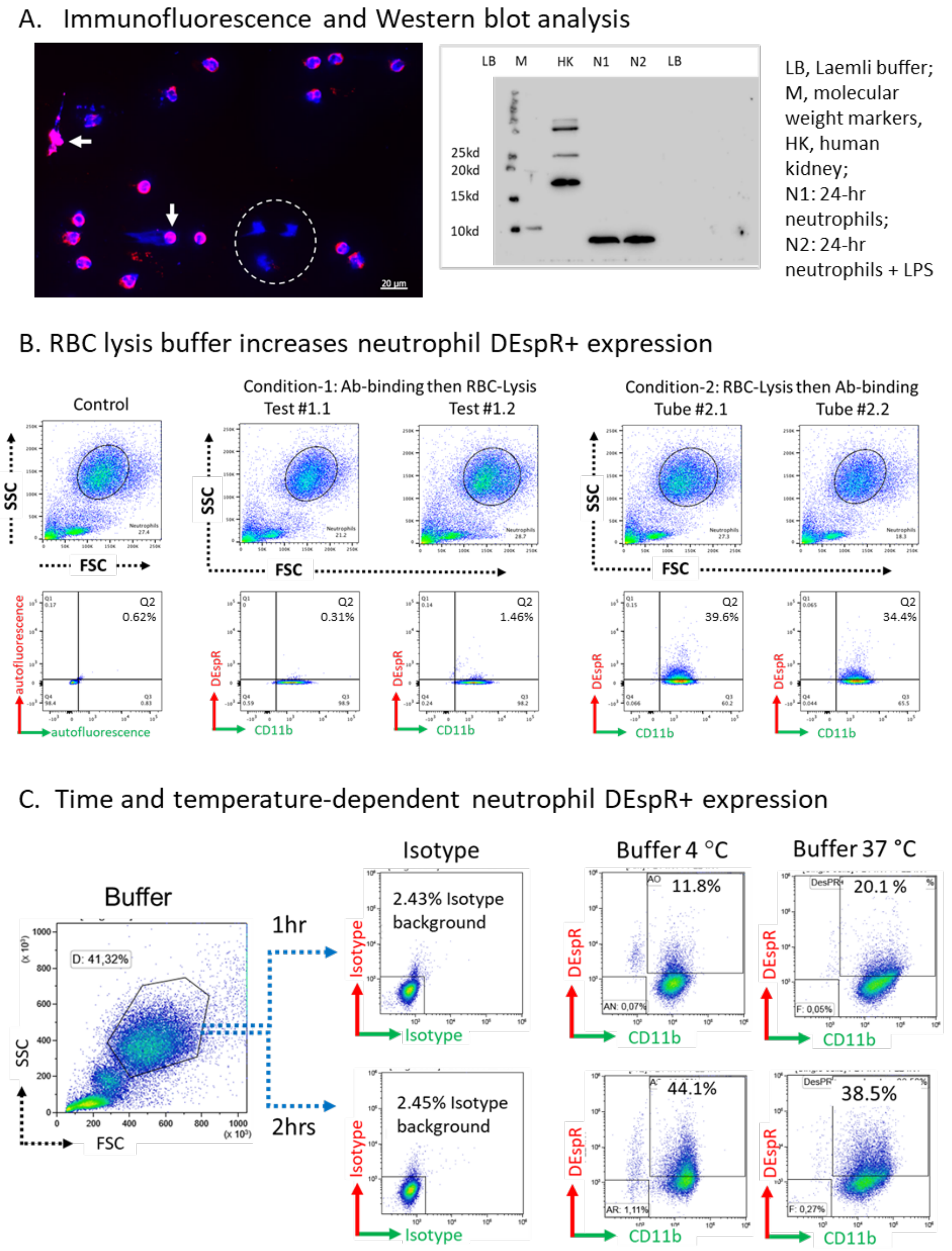

Supplementary Figure S1. Overview of DEspR+ expression in human neutrophils. (**A**) Left panel: representative immunofluorescence-staining of DEspR+ neutrophils, NETosing neutrophils, and DEspR(-) neutrophils in > 24-hour old normal human volunteer (NHV)-sample stored in 4°C RPMI buffer. Right panel: western blot analysis of human kidney (HK), and NHV neutrophils that have survived 24-hrs in cold storage (N1), and bacteriotoxin lipopolysaccharide (LPS)-stimulated NHV-neutrophils (N2). >24-hour survival stress approaches level of DEspR+ induction by LPS in neutrophils. (**B**) Representative flow cytometry (FCM) analysis of NHV neutrophils (EDTA-anticoagulated) shows effects of RBC-lysis procedure before or after binding of antibody for double immunophenotyping: anti-DEspR and anti-CD11b mAbs. (**C**) Representative FCM analysis of NHV neutrophils (Li-heparin anticoagulated) shows time- and temperature-dependent effects on NHV DEspR+ expression in *ex vivo* experimental system.

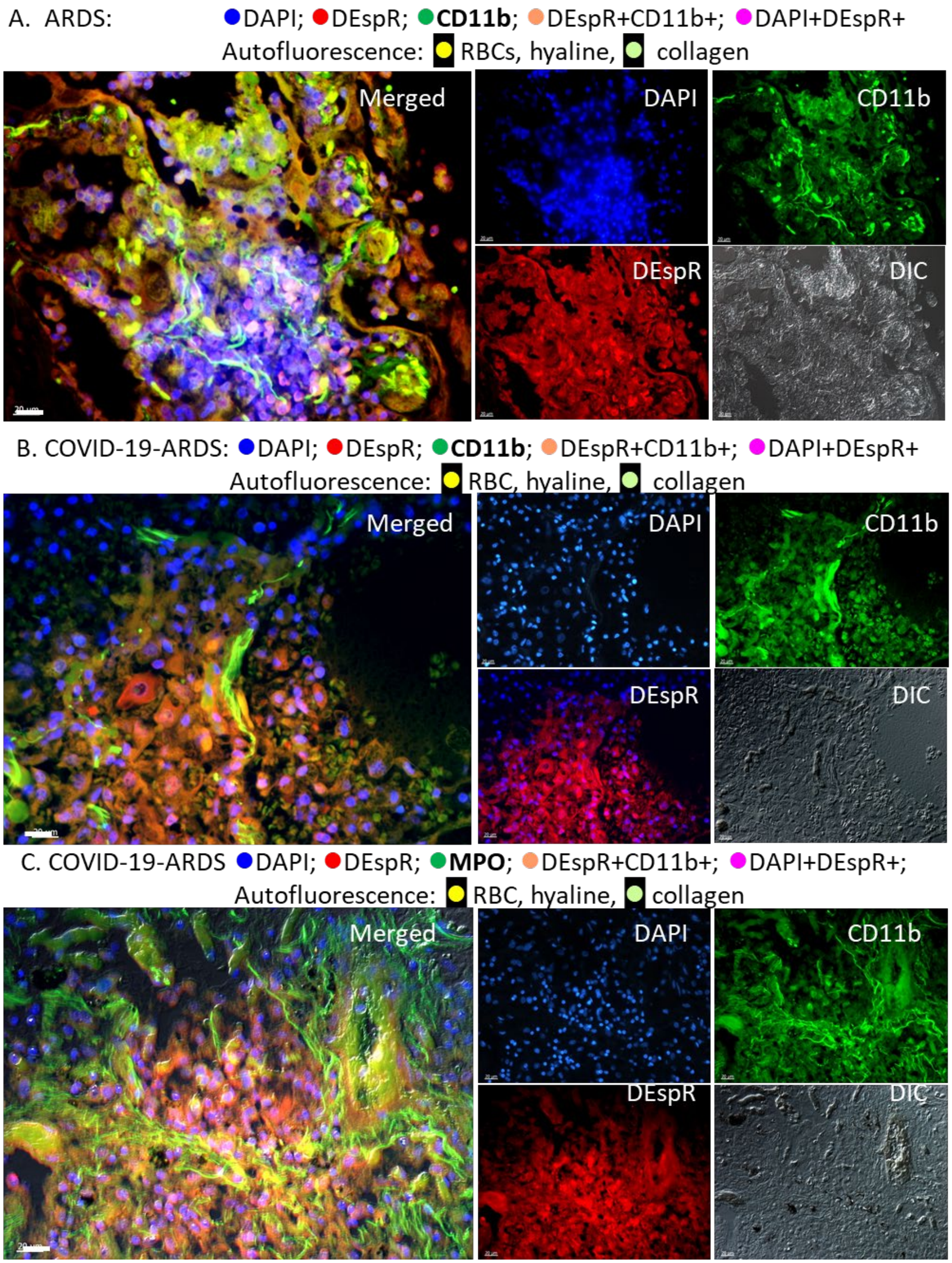

Supplementary Figure S2. Representative images of IF-stained DEspR+CD11b+ and DEspR+MPO+ post-mortem lung tissue sections from ARDS and COVID-19-ARDS patients.

(**A**) Representative DEspR and CD11b double IF-staining of post-mortem lung tissue-section from ARDS patient. (**B**) Representative DEspR and CD11b double IF-staining of COVID-19-ARDS lung tissue section. (**C**) Representative DEspR and MPO double IF-staining of COVID-19-ARDS lung tissue section.

Bar = 20 microns. Corresponding single fluorophore panels for DAPI, CD11b or MPO, and DEspR shown. DIC image of tissue section shown.

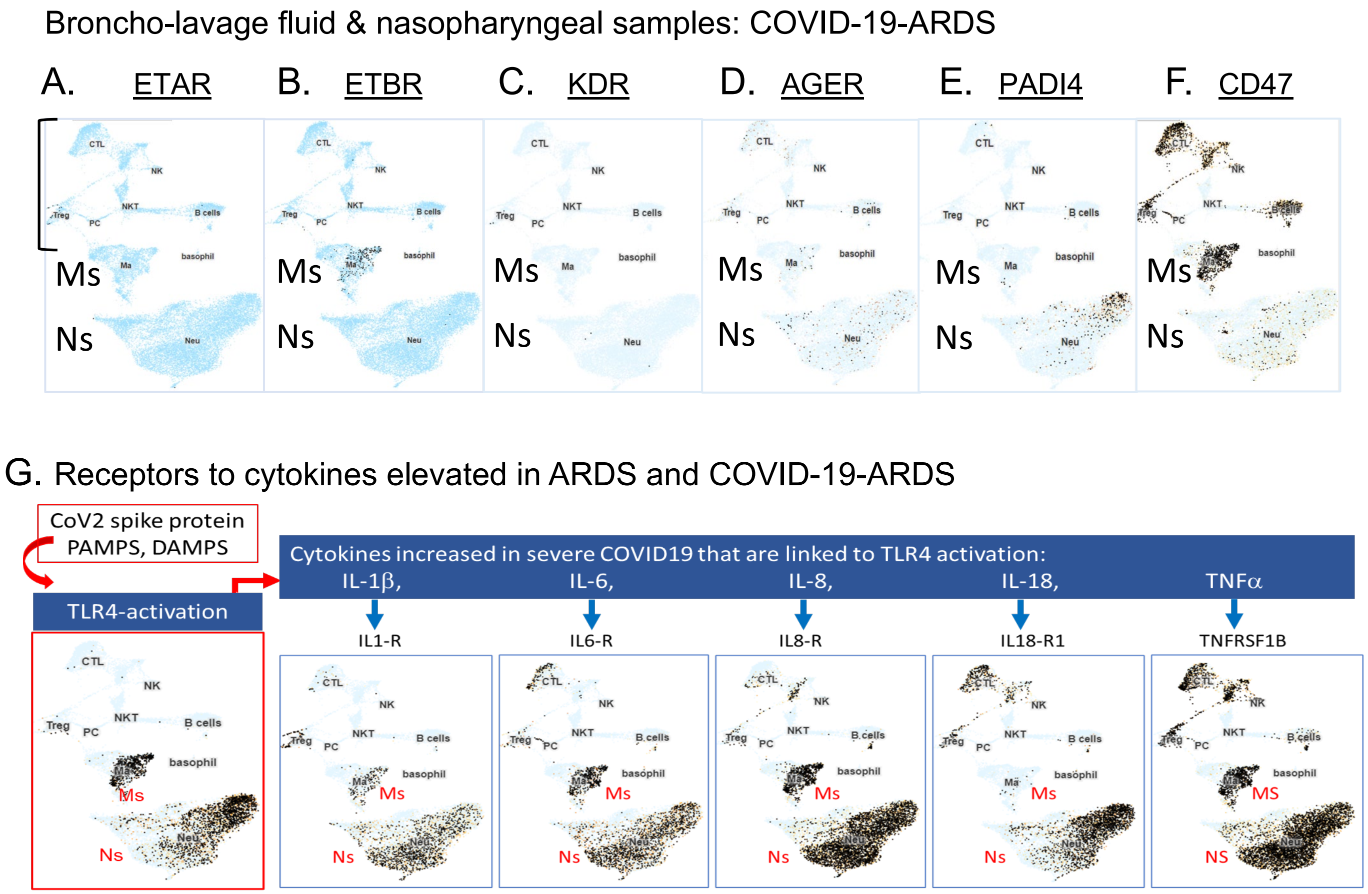

Supplementary Figure S3. Representative UMAP visualization of RNA-seq profiles of white blood cell clusters with > 2-fold increase (black dots) vs no expression (blue) obtained from broncho-lavage fluid and nasopharyngeal samples from two critically ill COVID-19-ARDS patients. (**A**) UMAP showing no expression of ETAR in neutrophils (Ns) and monocyte/macrophages (Ms); (**B**) UMAP showing no ETBR in neutrophils, few in monocytes; (**C**) UMAP of VEGF-R2 or KDR showing no expression; (**D**) minimal expression of AGER in neutrophils and monocytes; (**E**)**,** low expression of PADI4 in neutrophils, (**F**) minimal expression of CD47 'don't eat me' signal in neutrophils.

(**G**) scRNA-seq results show high levels of TLR4 in monocytes (Ms) and neutrophils (Ns), and presence of receptors for cytokines found to be elevated in ARDS and COVID-19-ARDS in both Ns and Ms. Greater expression in neutrophils is observed for IL-18 receptor. Diagram depicts direct activation of TLR4 on neutrophils and monocyte/macrophages via SARS-CoV2 (CoV2) spike protein docking on TLR4 as a pathogen-associated molecular pattern (PAMP), and/or from damage associated molecular patterns (DAMPs) such as alarmins S100A8, S100A9 released predominantly from high-expressing neutrophils (Fig. 3).

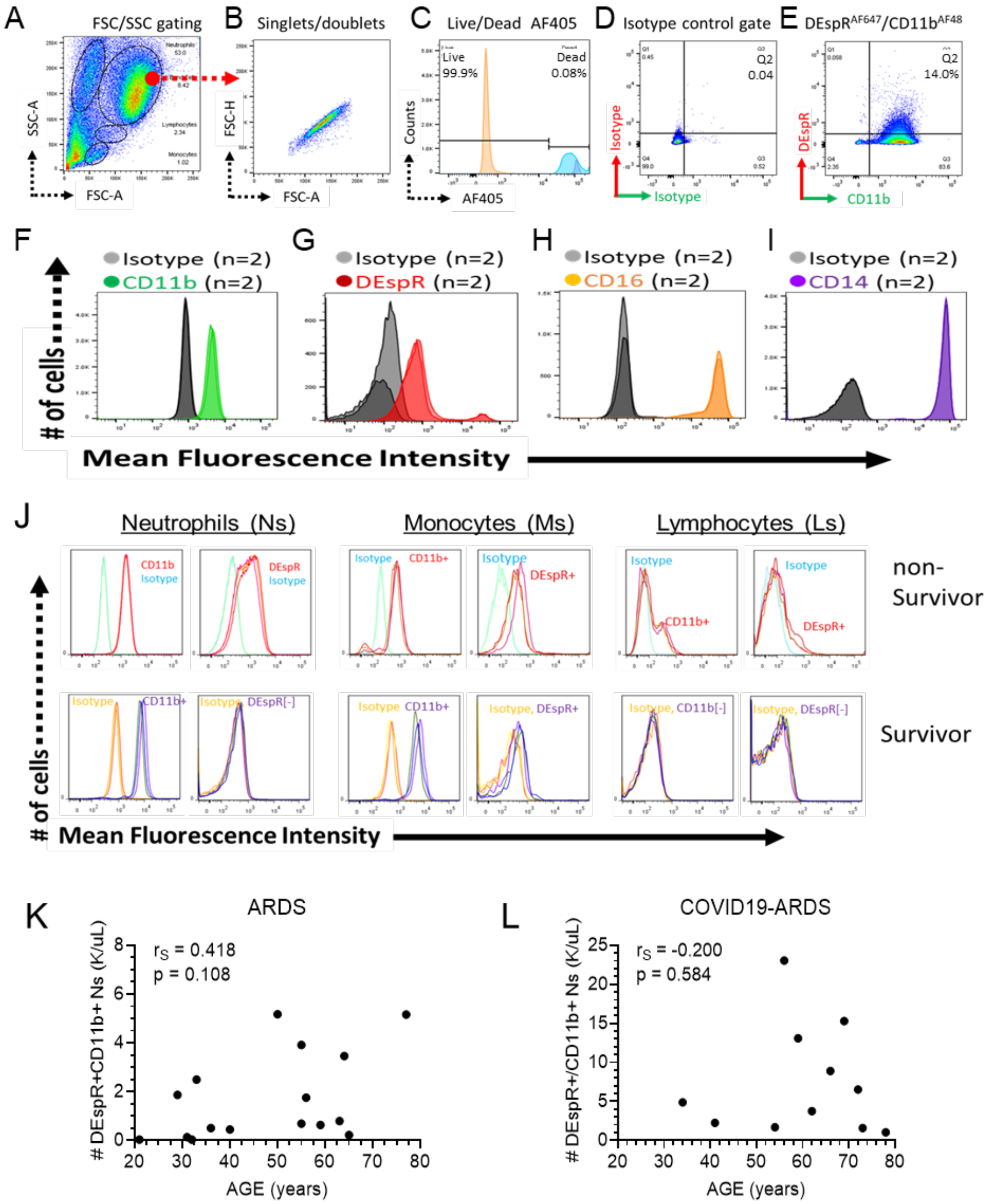

Supplementary Figure S4. Flow cytometry analysis of DEspR+ neutrophils. (**A**) Gating strategy for optical homogeneity or granularity (side scatter area SSC-A) and size (forward scatter area FSC-A), selection of neutrophil cloud (dashed ➔); (**B**) gating for singlets (FSC-A vs FSC-H); (**C**) affirmation of non-dead cells, (**D**) gating for isotype controls matched for isotype and fluorophore to both anti-DEspR and anti-CD11b antibodies; (**E**) representative scatter plot of DEspR+CD11b+ activated neutrophils in quadrant 2 (Q2); CD11b+DEspR[-] in Q3; CD11b[-]DEspR+ in Q1. Corresponding histograms of counts (# of cells) and Fluorescence intensity (x-axis) of antibody binding with matched respective isotype-fluorophores: (**F**) CD11b and isotype; (**G**) DEspR and isotype; (**H**) CD16 and isotype; (**I**) CD14 and isotype; all run in duplicates. (**J**) Histograms of mean fluorescence intensity in triplicate of CD11b+ and DEspR+ neutrophils, monocytes, and lymphocytes gated by FSC and SSC as shown in panel A. Representative flow cytometry results from ARDS non-survivor and ARDS-survivor. These MFI histograms correspond to triplicate dot plots of CD11b+DEspR+ immunotyping in Fig. 4A for neutrophils, monocytes and lymphocytes. (**K-L**) Scatter plot of number of DEspR+CD11b+ neutrophils (K/µL) vs age of patients showing no significant correlation (Spearman Rank Correlation coefficient (r_S) in patients with ARDS (n = 19) and in patients with COVID-19-ARDS (n = 11) requiring mechanical ventilation.

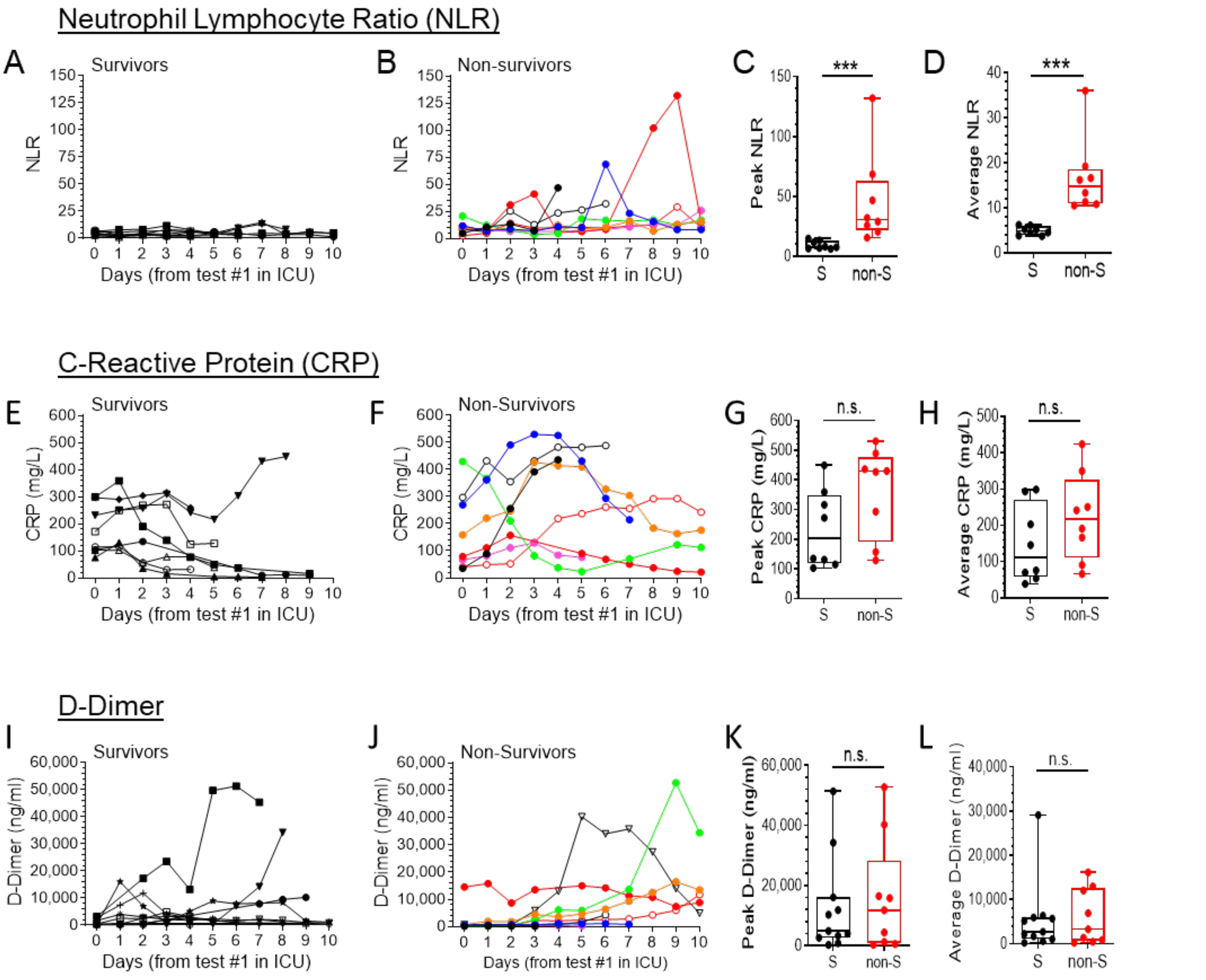

Supplementary Figure S5. Prospective analysis of biomarkers in patients with COVID-19-ARDS on mechanical ventilation: survivors (S: n = 8) vs non-survivors (nonS: n = 8). (**A-D**) Neutrophil lymphocyte ratio (NLR) analysis: time course while in the ICU (**A**) in survivors, (**B**) in non-survivors. Comparison of peak NLR (mean, 95% CI) in (**C**) survivors (9.37, 6.5-12.2) *vs* non-survivors (46.3, 14.1-78.4); (**D**) average NLR across ICU stay in survivors (mean, 95% CI: 4.9, 4.1-5.7) vs non-survivors (16.7, 9.7-23.7). Two tailed t-test P = 0.0002 (***) for C and D. (**E-H**) Analysis of C-reactive protein (CRP) levels: time course while in the ICU (**E**) in survivors, (**F**) in non-survivors; (**G**) comparison of peak CRP levels (mean, 95% CI) among survivors (235.0, 124.7-345.3) vs non-survivors (361.4, 235.1-487.7); and (**H**) average CRP levels comparing survivors (mean, 95% CI: 147.6, 58.7-236.5) vs non-survivors (222.8, 120.9-324.6). Two-tailed t-test did not detect significant difference in means for peak (G) or average (H) CRP (n.s.). (**I-L**) D-dimer levels: time course while in the ICU (**I**) in survivors (n = 11), (**J**) in non-survivors (n = 9); comparison graph of (**K**) peak D-dimer levels in survivors (mean, 95% CI: 12,699, 1861-23,537) *vs* non-survivors (15,944, 1574-30,314), and (**L**) average D-dimer levels in survivors (mean, 95% CI: 5533, 61-11,006) *vs* non-survivors (6035, 1269-10,801). Two-tailed t-tests detect no significant (n.s.) difference in means for both peak (K) and average (L) D-dimer levels.

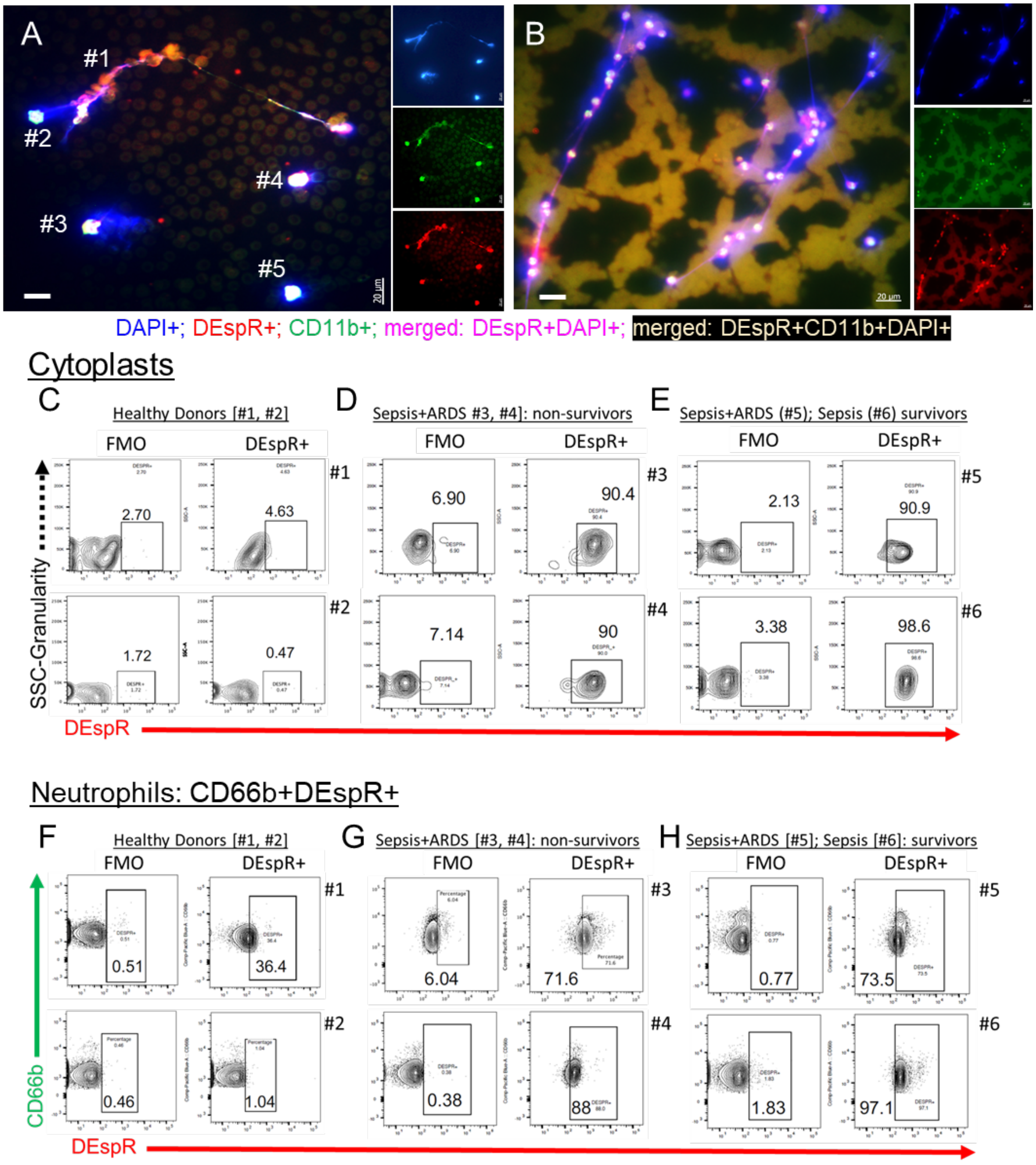

Supplementary Figure S6. Analysis of circulating DEspR+ neutrophil-extracellular trap (NET)-strands and cytoplasts. (**A**) Representative immunofluorescence staining of ARDS (non-COVID-19) patient blood smear shows #1: late/suicidal NETosing neutrophil with DEspR+CD11b+ NET-strand, #2, #3, #4: DEspR+CD11b+ early NETosing neutrophils with extruded DNA (DAPI+ but not DEspR or CD11b+); and #5: DEspR+CD11b+ non-NETosing neutrophil. (**B**) Representative immunofluorescence staining of COVID-19-ARDS NET-DNA strands that are DEspR+ and CD11b+, forming a beaded-string of DEspR+CD11b+ neutrophils or neutrophil-remnants. **(C-E)** Flow cytometry (FCM) analysis of low FSC/SSC DEspR+CD11b+ cytoplast remnants in (C) consented healthy donors (n = 2: 26y, 41y), (D) patients in the ICU with sepsis and ARDS (n = 3), or (E) sepsis-no ARDS (n = 1). White blood cells were isolated via 20-minute Inertial Microfluidic Separation validated without RBC lysis (see Methods) ~ 3 hours from blood draw. (**C**) Comparative FCM analysis of DEspR+ cytoplasts in healthy donors (sample #1, #2), compared with (**D**) non-survivors with sepsis-ARDS (#3, #4), and (**E**) survivors with sepsis-ARDS (#5) or sepsis-no ARDS alone (#6). (**F-H**) Using another marker of neutrophil activation representing degranulated neutrophils, CD66b, comparative FCM analysis of DEspR+CD66+ neutrophils in (**F**) healthy donors (sample #1, #2), compared with (**G**) sepsis-ARDS non-survivors (#3, #4), and with (**H**) survivor with sepsis-ARDS (#5) or sepsis-nonARDS (#6).

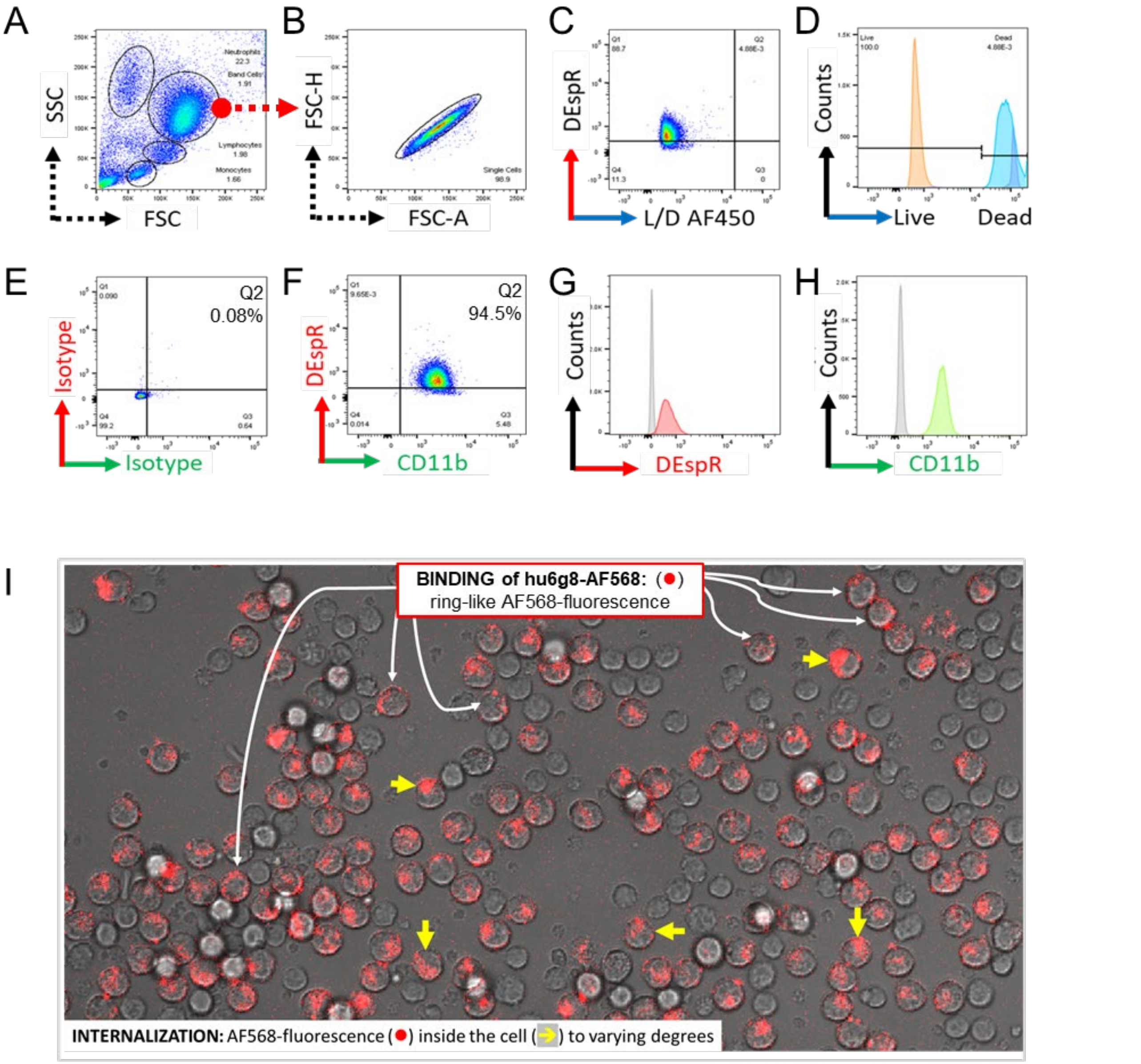

Supplementary Figure S7. Representative flow cytometry and live-cell images of DEspR+ NHP neutrophils. (**A-D**) Representative flow cytometry (FCM) analysis of NHP blood sample validating (**A**) gating of neutrophil cloud by FSC/SSC, (**B**) selection of singlets (FSC-A x FSC-H), (**C-D**) validation of DEspR binding to live cells with live/dead (L/D) AF450 stain dot plot (**C**) and histogram of mean fluorescence intensity distinguishing live *vs* dead cells (**D**). (**E-H**) Representative FCM analysis of NHP neutrophils. (**E**) Fluorescence gating based on matched isotype-fluorophore (hu-IgG4 AF647, mu-IgG2 AF488) controls, (**F**) DEspR+CD11b+ activated neutrophils are detected in 94.5% of all neutrophils, and confirmed on histograms of mean fluorescence intensities for (**G**) hu6g8-AF647, and (**H**) CD11b-AF488. (**I**) Representative confocal image at t-45 minutes after addition of hu6g8-AF568 10 µg/ml in PBS to NHP white blood cells, and incubated at 4 °C x 20 minutes, followed by washing off of excess unbound hu6g8-AF568 antibody, then placed into microfluidic chip. Live cell imaging begun at t-30 minutes documenting adhesion; image taken at t-45 minutes. White arrows (➔): hu6g8-AF568+ cell membrane, Yellow arrows (➔): cells with internalized hu6g8-AF568; DEspR+ (●).
